# Single Joint Hybrid Assistive Limb (HAL-SJ) robotic exoskeleton therapy in improving functional outcomes among workers with wrist fractures: Study protocol for a randomized controlled trial

**DOI:** 10.1371/journal.pone.0322191

**Published:** 2025-04-24

**Authors:** Eng Wah Tan, Siaw Chui Chai, Yoshiyuki Sankai, Masahiro Shingu, Nor Afifi Razaob, Hafez Hussain

**Affiliations:** 1 Occupational Therapy Programme, Centre for Rehabilitation & Special Needs Studies, Faculty of Health Sciences, Universiti Kebangsaan Malaysia, Kuala Lumpur, Malaysia; 2 PERKESO Rehabilitation Centre, Melaka, Malaysia; 3 Center for Cybernics Research, University of Tsukuba, Tsukuba, Ibaraki, Japan; 4 Cyberdyne Inc., Tsukuba, Ibaraki, Japan; Imperial College London, UNITED KINGDOM OF GREAT BRITAIN AND NORTHERN IRELAND

## Abstract

**Introduction:**

Robotic technologies have been developed for motor rehabilitation and such robots have shown favourable results when compared with equivalent doses of usual clinical therapy. Recently, robotic interventions have been suggested to be applied in orthopaedic rehabilitation with upper extremity disorders, especially those related to hand and wrist. This study aims to determine the effectiveness of combined conventional therapy and HAL-SJ robotic therapy in restoring the wrist functionality following the fractures as compared to the standard conventional therapy solely.

**Methods and analysis:**

Workers with wrist fractures will be randomized in two groups, i.e., the control group (conventional therapy) and intervention group (combination of conventional therapy and robotic HAL-SJ intervention). All participants will receive 5-day/week therapy sessions for four weeks. Primary outcomes of the Disabilities of the Arm, Shoulder and Hand (DASH) outcome measure and secondary outcomes of range of motion, grip and pinch strengths, fine and gross hand dexterity as well as pain and the Lam Assessment of Stages of Employment Readiness (LASER) will be assessed at baseline assessment and upon completion of the therapy program after 4 weeks. Data from the baseline and post intervention outcome measures will be analysed using a Repeated Measures ANOVA to compare the therapy effectiveness of both control and intervention groups.

**Results:**

Participants recruitment and data collection are in progress

**Discussion:**

Wrist fractures can produce some residual disability and pain that may impact the functionality of a person. The application of robotic technology in facilitating upper limb movement and functional recovery training is extensive and shows positive outcomes in the field of neurorehabilitation. However, there is a lacking of published evidence about the effectiveness of robotic intervention in orthopaedic rehabilitation, especially in the field of hand therapy.

**Conclusion:**

Participants recruitment and data collection are still ongoing

**Clinical trial registration:**

This trial is registered with the Australian New Zealand Clinical Trials Registry (registration number: ACTRN12622000413729).

## Introduction

Work-related hand injuries contributed to 24.9% of all industrial accidents seen at the emergency departments and orthopaedic clinics in Malaysia [1]. Approximately 98.6% of workers with traumatic hand injuries returned to work after a median absence of 45.5 days [2]. Hand injury costs had brought cumulative economy impact not only to patients, but their family, employer, and society [3].

Decreased wrist flexion and/or extension after trauma or surgery can be a challenging problem [4]. A study in UK reported that majority of patients had some degrees of disability one year after distal radius fracture with 16% had moderate to very severe disability and were not working [5]. In addition, patients who have high self-reported pain/disability and occupational demand at baseline have an average of 9.2 weeks of work loss following distal radius fracture [6].

The goal of restoring wrist motions is to reduce impairments and enhance functional performance for activities of daily living, work, and leisure. Improper rehabilitation program after wrist injury may significantly impair the overall upper extremity function [7]. A study by MacDermid et al. [6] indicates that both injury and patient factors can impact the management and outcomes after distal radius fracture. Although optimal physical parameters, including strength, range of motion (ROM), and function are achievable within 3–6 months of conservative or surgical management, some patients require longer duration to complete the rehabilitative phase.

With respect to the employability issues of the workers with wrist fractures, the advocation of matching treatment to the patient’s stage of employment readiness appears to be a more effective approach to service provision [8]. A timely and goal-oriented treatment-matching approach in the work program will deliver better outcomes in term of cost effectiveness, employment, and service quality.

Robotic technologies developed for motor rehabilitation have shown to produce favourable results when compared with equivalent doses of usual clinical therapy [9,10]. Compared to manual therapy, robotic intervention can potentially provide greater impact on impairment due to easy deployment, applicability across of a wide range of motor impairment, high measurement reliability, and the capacity to deliver high dosage and high intensity training protocols for a longer duration, irrespective of the skills, and fatigue level of the therapist [11]. Given the current concepts of robotic intervention are focused on providing motor control/learning, practice-induced neuroplasticity, intensity, and task specific training, the application of robotic intervention in upper limb orthopaedic rehabilitation, especially concerning the hand and wrist has been suggested [12].

The Single Joint Hybrid Assistive Limb (HAL-SJ) is one of the robotic devices that can be used for upper limb rehabilitation. The Hybrid Assistive Limb (HAL) represents a cyborg-type robot designed to amplify, enhance, and assist physical abilities, thereby advancing and elevating human capabilities through the cutting-edge science of “Cybernics”. Cybernics constitutes a burgeoning interdisciplinary field focusing on the integration of neuroscience, robotics, systems engineering, information technology, “kansei” engineering, ergonomics, physiology, social science, law, ethics, management, economics, and more, within the realms of cybernetics, mechatronics, and informatics [13]. The newly developed wrist joint attachment to the HAL-SJ can assist voluntary articulatory movements of the wrist joint including wrist flexion and extension, and forearm supination and pronation. Investigating the outcome of HAL-SJ among upper limb injured workers, especially those with wrist fractures shall provide further evidence on the effectiveness and feasibility of robotic interventions in the field of hand therapy and upper limb rehabilitation.

### Objectives

The primary objective of this study is to determine the effectiveness of Single Joint Hybrid Assistive Limb (HAL-SJ) robotic therapy in improving functional level, physical performance, and work readiness among workers with wrist fractures after 4 weeks of intervention.

### Hypotheses

A 4-week combined conventional therapy and Single Joint Hybrid Assistive Limb (HAL-SJ) robotic therapy provides greater functional and impairment levels improvement among workers with wrist fractures as compared to those receiving conventional therapy alone during the same intervention period.

## Method

### Research design

**Fig 1:**
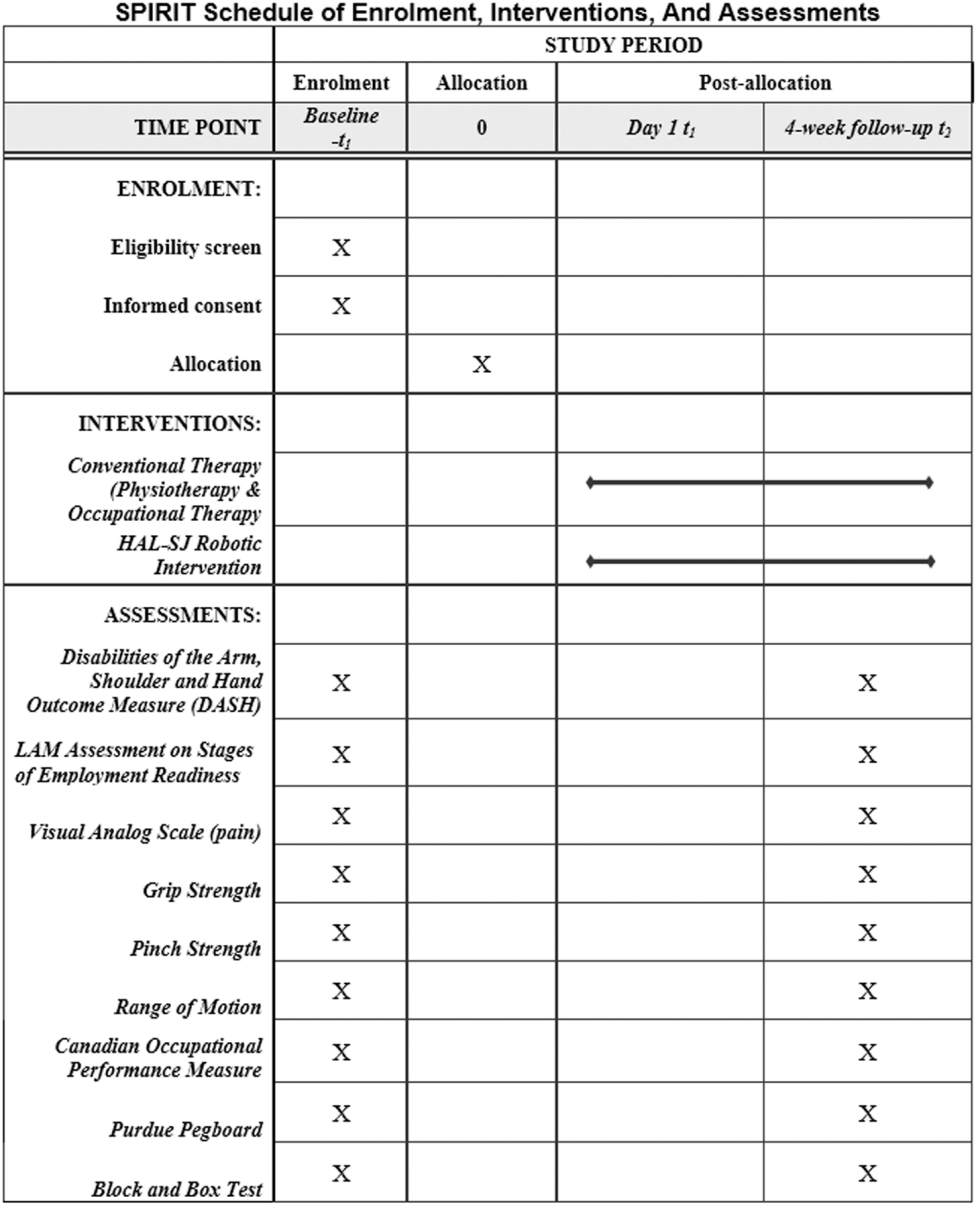
SPIRIT schedule.

**Fig 2:**
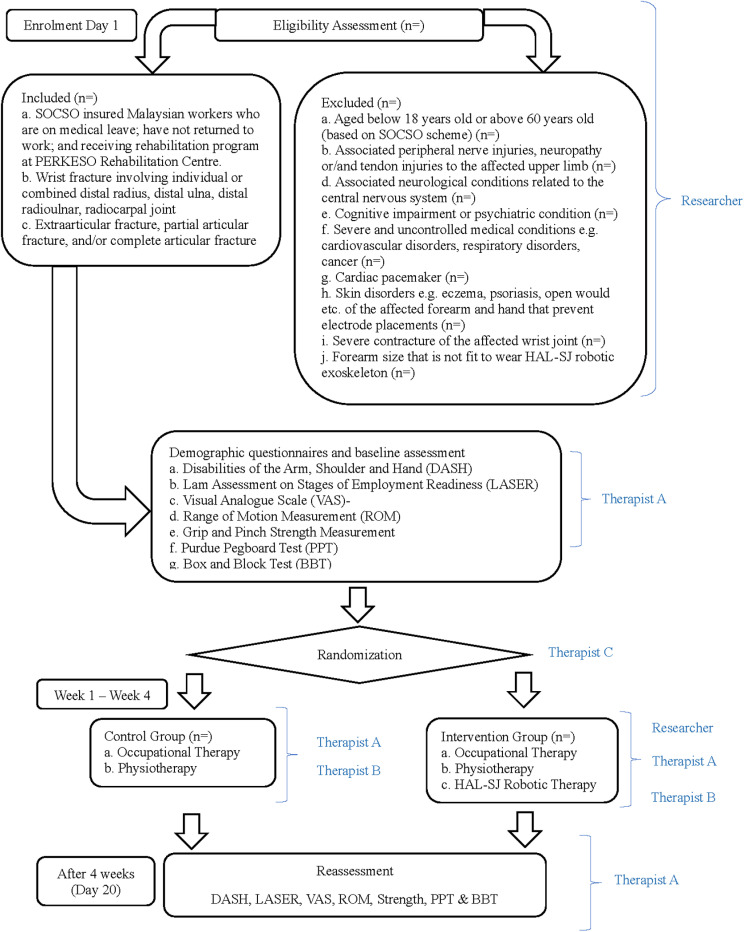
Study flow.

This is a randomized controlled trial (RCT) design where the effects of the study treatment (intervention) are compared with those of a control treatment after the patients were being randomly assigned to the two groups, i.e., the control group and the intervention group. The protocol for this study is guided by the Standard Protocol Items: Recommendations for Interventional Trials (SPIRIT) checklist consisting of a 33-items. The checklist for this study is detailed in S1 File. The SPIRIT Schedule of Enrolment, Interventions, and Assessment is shown in Fig 1 while the flow for this study is detailed in Fig 2 beginning with participant enrollment, group allocation, baseline assessment, intervention and ending with reassessment. The study protocol with information sheet, consent form, demographic questionnaire, and data collection form are included in the S2 File.

### Participants: Recruitment and eligibility criteria

The target participants for this study are Social Security Organisation (SOCSO) insured workers with wrist fractures who meet both the inclusion and exclusion criteria and are receiving rehabilitation management at PERKESO Rehabilitation Centre. The inclusion criteria are wrist fractures affecting individual or combined distal radius, distal ulna, distal radioulnar, and/or radiocarpal joint, as well as extraarticular fracture, partial articular fracture, and/or complete articular fracture. The exclusion criteria are: associated peripheral nerve injuries, neuropathy, and/or tendon injuries to the affected upper limb; associated neurological conditions related to the central nervous system; cognitive impairment or psychiatric conditions; severe and uncontrolled medical conditions such as cardiovascular disorders, respiratory disorders, cancer; skin disorders such as eczema, psoriasis, open wounds, etc., of the affected forearm and hand that prevent electrode placements; severe contracture of the affected wrist joint; and those with forearm size that is not suitable for wearing the HAL-SJ robotic exoskeleton. The recruitment period for this study has been started on 21 June 2021 and will end by 20 June 2025.

### Sampling size

The sample size for each hypothesis was calculated using G*Power analysis. Since this is a novel study without reference from previous research, the sample size was determined using ANOVA repeated measures, within-between interaction, with a Cohen’s f effect size set at 0.25 for a medium effect, an alpha error of 0.05, and 80% power. The results indicated that a total of 34 participants would be required. Factoring in a 10% dropout rate, the necessary sample size increased to 38 participants with 19 participants allocated to the interventional group and 19 participants to the control group.

### Randomization

All patients with wrist fractures referred to PERKESO Rehabilitation Centre will be randomly assigned to the control and intervention groups by Therapist C, an independent therapist who is not involved in the assessment or intervention process. Simple randomization with allocation concealment will be used to prevent the researcher from influencing which participants are assigned to the intervention group. After completing the baseline assessment process, each participant will receive a sealed opaque envelope containing an equal number of random allocation cards designated with the alphabet “C” (Control group) and “I” (Intervention group). Therapist C will record the group allocation for each participant. Selected participants will be informed about the study and included only after giving their informed consent.

### Blinding

The baseline assessment of each participant will be conducted by Therapist A before the randomization process to maintain the blinding of the respective therapists and participants. The researchers will not participate in any baseline or outcome assessment processes throughout the study. The group allocation for each participant will be revealed upon the completion of the randomization process by Therapist C.

### Research team

This study involves various clinicians and administrative staff from the PERKESO Rehabilitation Centre, including the Researcher (primary author EWT), Therapist A (an Occupational Therapist), Therapist B (a Physiotherapist), Therapist C (an independent therapist), and a case manager. All patients with wrist fractures referred to the PERKESO Rehabilitation Centre will be categorized through the admission database by the case manager. The Researcher will then conduct eligibility screening for each participant based on the inclusion and exclusion criteria. As a certified HAL-SJ operator accredited by Cyberdyne Inc., the Researcher will assume the role of delivering the robotic intervention for participants in the intervention group. Therapist A will serve as the baseline and outcome assessor and will conduct conventional occupational therapy interventions. Prior to group allocation through the randomization process by Therapist C, Therapist A will perform baseline assessments for all enrolled participants. Meanwhile, Therapist B will be responsible for conducting the conventional physiotherapy interventions outlined in the study (Fig 2).

### Informed consent, registration, baseline assessment and group allocation

The study flow will be elucidated to the selected participants upon confirmation of their eligibility for the study. An information sheet (S2 File) detailing general information, purpose, procedures, risks and benefits, as well as data confidentiality, will be provided and explained to each participant. Participants will only be included in the study after signing the informed consent forms. Following this, baseline assessment and collection of demographic data for each participant will be conducted by Therapist A. Subsequently, participants will be allocated into either the control or intervention group by Therapist C through simple randomization with allocation concealment (Fig 2).

### HAL-SJ robotic exoskeleton

**Fig 3:**
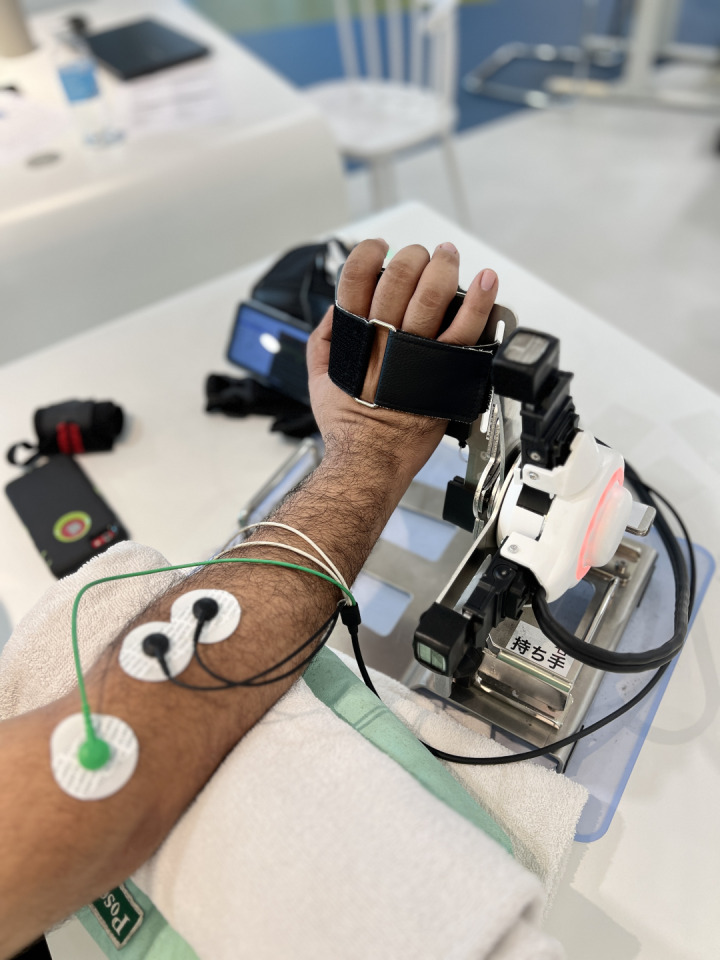
Single Joint Hybrid Assistive Limb (wrist extension).

**Fig 4:**
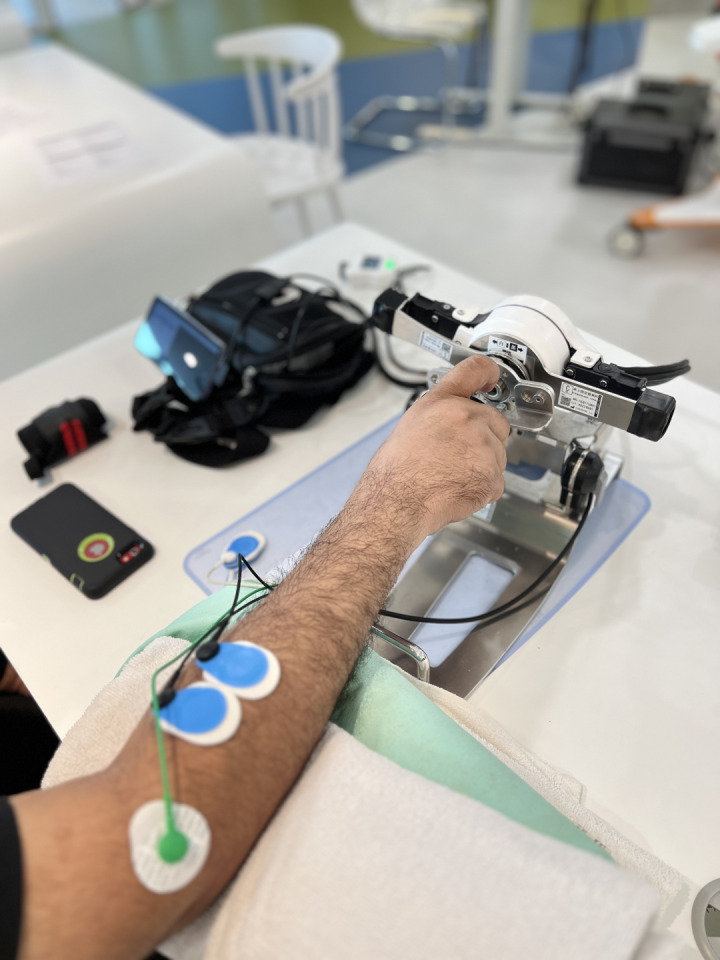
Single Joint Hybrid Assistive Limb (forearm rotation).

HAL-SJ robotic exoskeleton is certified by ISO 13485:2016 (certificate number: 1757.181211) and EC (registration number: DD 601417310001) for rehabilitation and physical therapy usage. The upper limb HAL-SJ is a wearable movement support robot that detects bioelectrical signals on the skin surface and assists joint movements by controlling and operating an actuator placed outside the respective joint. The power units of HAL generate power assist torque by amplifying the wearer’s own joint torque estimated from his/her bioelectrical signals, and the support motions are consequently controlled. It is a safe device that can be used for rehabilitation intervention as approved by the FDA, EC and MDA. It may cause several minor problems: (a) skin allergy or reddening of area where electrode is affixed, however, the reddening should disappear shortly after the electrode was taken off; (b) abrasion of areas that contact the device, e.g., cuffs and straps; and (c) muscle and joint soreness (due to post exercise effect). The researcher of this study, who is a certified HAL-SJ operator accredited by Cyberdyne Inc. will take necessary precaution and careful supervision throughout the entire HAL-SJ robotic exoskeleton session to minimize these problems. The HAL-SJ robotic exoskeleton that will be used is shown in Figs 3 and 4.

### Study protocol

The design of this study is an interventional, parallel, superiority randomized clinical trial on a consecutive convenience sample of 38 participants using 1:1 ratio. The study protocol involves a combination of conventional therapy and HAL-SJ robotic therapy. Each participant from both the control and intervention groups will undergo two conventional therapy sessions daily, consisting of an occupational therapy session and a physiotherapy session, on a 5-day/week basis. Each occupational therapy and physiotherapy session will last for 90 minutes, respectively. Additionally, the intervention group will receive a 60-minute HAL-SJ robotic therapy session conducted by the researcher daily, following the conventional therapy sessions. All participants will receive these 5-day/week therapy sessions for a duration of 4 weeks.

### Study protocol for conventional therapy

Both the control and intervention groups will undergo conventional therapy, which comprises occupational therapy and physiotherapy interventions aimed at wrist fracture rehabilitation. This therapy encompasses various interventions such as pain management, range of motion exercises, progressive strengthening exercises, functional activities training, as well as work conditioning and work hardening activities.

In the initial week, the interventions prioritize restoring joint mobility and pain management. Occupational therapy interventions during this phase encompass active and passive ROM exercises for the digits, elbow, and shoulder, active ROM exercises for the wrist and forearm rotation, tendon gliding exercises, and dexterity exercises. Physiotherapy interventions involve joint mobilization, soft tissue mobilization, retrograde massage, heat/cold modalities, ultrasound therapy, and electrical stimulation.

The conventional therapy program from the first week will be sustained into the second week of intervention, with the inclusion of functional activities training from occupational therapy. Additionally, physiotherapy will introduce isometric and isotonic strengthening exercises during this phase.

During the third week, the focus shifts to a graded strengthening program. This includes isometric and isotonic strengthening exercises, progressive resisted exercises, as well as open and closed kinetic chain exercises. All interventions conducted during the first and second weeks will be maintained throughout the third week of the conventional intervention phase.

In the fourth week of the conventional therapy program, interventions will involve work simulation, work conditioning, and work hardening activities. All interventions from previous weeks will be continued based on the individual’s condition.

### Study protocol for HAL-SJ Robotic Therapy

The HAL-SJ Robotic Therapy protocol will utilize the gentle mode of the HAL-SJ exoskeleton It comprises four phases that will progress over the 4-week intervention period, adjusting according to four parameters: assist gain, assist level, flexion/extension signal balance level, and active/relax phase duration. Each intervention session will include four types of wrist exercises: wrist extension, flexion, supination, and pronation. The protocol entails three sets of exercises, with 50 repetitions for each specific wrist movement (extension, flexion, supination, and pronation), performed with an active/relax phase duration of 10 seconds/10 seconds.

The assist level will remain constant at x1 level throughout the 4-week intervention period. However, the assist gain and the flexion/extension balance signal will be adjusted based on the individual’s progress from the first week to the fourth week of the intervention period.

During the first week, the intervention will be carried out with the highest assist gain set at 75–100%, while maintaining the assist level at x1. Simultaneously, the flexion/extension balance signal level will be maintained at 100% flexion/100% extension. In the second week of the intervention, the assist gain will be reduced to 50–75%, while the flexion/extension signal balance level remains at 100% flexion/100% extension. As the intervention progresses into the third week, the assist gain will be further reduced to 25–50%, while the flexion/extension signal balance level will be adjusted based on the desired wrist exercise movements. During wrist extension or supination, the flexion/extension signal balance level will be set at 50% extension (supination)/100% flexion (pronation), and vice versa. In the fourth week of intervention, the assist gain will be set to the lowest level at 0–25%. During this phase, the flexion/extension signal balance level will be regulated to 25% for the desired wrist movement while the remaining 100% signal will be adjusted for the antagonistic movement.

### Outcome measures

The primary outcome measure is Disabilities of the Arm, Shoulder and Hand (DASH) Outcome Measure for gauging the upper extremity functional improvement. The DASH assesses physical function and symptoms for various musculoskeletal conditions of the upper extremity [14]. It has extensively researched psychometric properties [15] with validity and responsiveness in both proximal and distal disorders [16]. Focusing on disability level, each item has five response options and total score can be computed using a provided formula, i.e., total score = ([(sum of n responses)/n]-1)×25 with a higher score indicating greater disability [17].

There are 6 secondary outcomes. Firstly, ROM, including both active and passive ROM of the forearm, wrist and fingers are assessed. Assessment begins with wrist flexion, extension, radial deviation and ulnar deviation as well as supination and pronation using a wrist goniometer. This is followed by assessments on the same wrist and forearm joints to evaluate the end feel. For fingers, active and passive ROM of proximal interphalangeal joint, and distal interphalangeal joint are assessed using a finger goniometer. Grip and pinch (lateral pinch, tripod pinch, and tip-to-tip pinch) strengths are secondary outcomes measured using the respective Jamar (using a dynamometer (handle 2) and B&L Engineering Pinch Gauge measured three times using a position recommended by the American Society of Hand Therapists [18].

Another secondary outcome measures are fine and gross hand dexterity as measured using the Purdue Pegboard Test (PPT) and the Box and Block Test (BBT) respectively. The PPT is a valid and reliable standardized instrument with five subtests. The first three subtests involve placing the maximum number of pins into holes on the pegboard using the dominant hand, the non-dominant hand, and then both hands simultaneously, all within a 30-second time frame. The fourth is not an actual test but involves a mathematical calculation. The fifth test involves using alternate hands to assemble components including pins, collars, and washers, within a 60-second timeframe [19]. Meanwhile, the BBT is widely used across various clinical populations due to its ease and efficiency of implementation [20,21]. It involves transferring 125 2.5 cm³ wooden blocks, arranged in various orientations, from one side of a partition to another using the testing hand as quickly as possible within a 1-minute timeframe. The participant’s score corresponds to the number of blocks successfully transported over a 15.2-cm tall partition in one minute [20,21]. It has high inter-rater and test-retest reliability, with intraclass correlation coefficients ranging from 0.85 to 0.97 [22–24]. Normative data for the BBT in adults were published in 1985, based on a sample of 628 individuals without disabilities spanning ages 20–70+ years [20].

Pain is a secondary outcome measured using Visual Analogue Scale (VAS). It is a single-item tool that captures the entire pain construct at once. Consisting of a 100mm horizontal line with two opposing labels, “no pain” and “severe pain,” participant indicates his/her pain level by marking a score along the scale using a vertical line. Due to its ease of use, the VAS is applicable across a variety of practice and research settings [25].

Readiness to work as measured using the Lam Assessment on Stages of Employment Readiness (LASER) is a secondary outcome of this study. LASER is a common instrument used to assess the psychological readiness of workers to return to work following an extended period of unemployment due to disability [8,26]. LASER comprises 14 items aligned with Prochaska’s stages of change model, delineating behaviors within three stages of work readiness, i.e., the pre-contemplation (6 statements), contemplation (4 statements), and action stages (4 statements). The different sub-scores representing the corresponding stages, forming a continuous measure. The highest sub-score reflects a subject’s inclination towards the corresponding stage. Subjects in the pre-contemplation (PC) stage do not perceive unemployment as an issue and may lack interest in working or believe they are unable to work. Subjects in the contemplation (C) stage begin weighing the pros and cons of working but haven’t taken any action such as job searching yet. Subjects in the action (A) stage have made the decision to work by actively seeking employment and addressing many barriers related to returning to work and they would have the best employment outcomes [8,26].

Given that outcome measures commonly employed for wrist fractures include wrist range of motion (ROM) measurement, grip/pinch strength measurement (using a dynamometer and pinch gauge), pain measurement (Visual Analogue Scale), and the Disabilities of the Arm, Shoulder and Hand (DASH) Outcome Measure [27], all of these outcome measures will be utilized in this study. Additionally, both the Purdue Pegboard Test and Box and Block Test will be employed to assess functional performance in terms of hand dexterity, while readiness to work will be evaluated using the LAM Assessment on Stages of Employment Readiness (LASER) Questionnaire. All the baseline and outcome assessments will be conducted by Therapist A who is an experienced occupational therapist in the field of hand therapy.

### Statistical analysis

Data will be analyzed using the IBM® SPSS® Statistics version 29. Descriptive statistics for categorical data, including gender, marital status, hand dominance, types of medical management and intervention, type of wrist fractures, educational level, employment status, and type of SOCSO compensation scheme, will be expressed in frequency and percentage. Descriptive statistics for continuous data, including age, the scores of a primary outcome measure of DASH, and the secondary outcomes measures, including ROM, grip and pinch strength, pain, hand dexterity, and work readiness, will be used expressed in mean, standard deviation, maximum, and minimum if the data is normally distributed or in median and inter-quartile range if the data is not normally distributed. The Shapiro-Wilks test will be used to test the normality of the continuous data. All statistical tests will be performed using two-sided tests at the 0.05 significance level.

A Repeated Measures ANOVA will be used to analyze the mean differences in treatment outcomes within and between the groups at baseline and after 4 weeks of intervention. ANOVA assumes that the data in each group are normally distributed and that the variances are equal across groups (homogeneity of variance). If the data is not normally distributed, a transformation will be applied, including log, square root, or Box-Cox transformation. If the data cannot be transformed to meet ANOVA assumptions, a non-parametric alternative, i.e., the Kruskal-Wallis test, will be conducted.

A Repeated Measures ANOVA is used for independent observations in this study by comparing the means across the independent groups, i.e., control and intervention groups. With respect to this, the data in this study is balanced where it has equal sample sizes across the control and intervention groups. A Repeated Measures ANOVA compares the mean differences between groups that have been split on two “factors” (also known as independent variables), where one factor is a “within-subjects” factor and the other factor is a “between-subjects” factor. For example, in this study, a Repeated Measures ANOVA is used to measure a dependent variable (functional outcome) over two time points (baseline and after 4 weeks of interventions), i.e., where “time” is the “within-subjects” factor), but also when the subjects (participants) have been assigned into two separate groups when they undergo difference interventions (i.e., the control and intervention group). These groups form the “between-subjects” factor. The within-subjects factor is time while the between-subjects factor consists of the types of interventions. The primary purpose of a Repeated Measures ANOVA is to understand if there is an interaction between these two factors on the dependent variable. Since we are using the ANOVA for analysis, we do not include random effects where it treats all groups as fixed effects.

With respect to Repeated Measures ANOVA analysis, the independent factors in this study are the combination of robotic intervention with conventional therapy and the conventional therapy alone with DASH scores (primary outcome) and all six secondary outcomes act as an dependent variable, respectively. This study will assess the main effects of time and group and their interaction effect. The change in the pre- and post-functional level (DASH scores) (main effect 1) among workers with wrist fractures between the intervention and control groups (main effect 2) will be compared and analyzed. The study will also explore the difference in functional level between pre- and post-interventions in both control and intervention groups (interaction effect).

Other key assumptions will be assessed, i.e., sphericity and homogeneity of variances. The Mauchly’s test of sphericity will be used to determine whether the variances of the differences between all pairs of levels of a within-subjects factor are equal. If the p-value is significant (p < 0.05), the assumption of sphericity is violated. If sphericity is violated, the degrees of freedom of the F-statistic will be adjusted using correction Greenhouse-Geisser or Huynh-Feldt corrections, making the test more conservative [28]. For the homogeneity of variances, Levene’s test will be used to evaluate whether the variances of the dependent variable are equal across groups. If the p-value is significant (p <.05), it indicates a violation of the assumption of homogeneity of variances [29,30].

The results will be expressed as mean differences between groups, along with their corresponding two-sided 95% confidence intervals. Statistical significance will be determined by p-values, which will be reported to three decimal places. For p-values less than 0.001, the notation “<0.001” will be used.

This study will adopt the principle of intention-to-treat (ITT) analysis. The ITT principle is a cornerstone in RCT analysis, emphasizing that all randomized participants in an RCT should be analyzed according to their assigned treatment group, regardless of treatment completion, discontinuation, or adherence to the study protocol. This process has once been described as “once randomized, always analyzed” [31–33].

Standard statistical methods for analyzing RCT data typically incorporate baseline measurements as covariates. However, when a participant has a recorded baseline measurement but is missing all subsequent follow-up data, the ITT principle implies that they should still be included in the analysis based on their assigned group. This poses a methodological challenge that requires careful consideration [32]. A common challenge in clinical trials is evaluating data from patients who drop out or cannot complete the full study [32,34–38].

If there is any missing data occurrence happened, this study will use the baseline observation carried forward (BOCF) method to handle patient dropouts. The BOCF uses the baseline observation and handles missing data from early treatment discontinuation. For participants who discontinued participation in the trial, their baseline observation will be considered their final response, irrespective of the reason for withdrawal or their scores at the time of withdrawal. For participants who completed the intervention, the BOCF endpoint was their last recorded observation [35]. This method has been proposed as a more reliable and preferred imputation strategy over the last observation carried forward (LOCF) method [35,36,39]. The LOCF method may yield biased estimates of treatment effects and compromised statistical tests of the null hypothesis of no treatment effect [40]. The LOCF method is also prone to substantial misrepresentation of trial results and is an unsuitable choice for primary analysis [41].

Besides, it is essential to consider both treatment-related and non-treatment-related reasons for dropout in the BOCF approach. Treatment-related reasons for discontinuation comprised adverse events and insufficient efficacy. Non-treatment-related reasons encompassed failure to meet inclusion criteria, protocol deviations, loss to follow-up, patient-initiated withdrawal (e.g., work-related conflicts, transportation difficulties), and investigator-initiated withdrawal (e.g., site closures, unreliable patient behavior) [35].

### Monitoring, ethics and dissemination of information

The study has obtained ethical approval from the Research Ethics Committee of Universiti Kebangsaan Malaysia (JEP-2021–201) and the Scientific Committee of PERKESO Rehabilitation Centre (PRPTAR.600-5(48)). Besides, it has been registered at the Australian New Zealand Clinical Trials Registry (ANZCTR) with registration number: ACTRN12622000413729.

Participation in this study is entirely voluntary, and individuals have the right to withdraw from the study at any point without facing any pressure or coercion. Each participant will receive an information sheet detailing the study. Those who choose to participate will be provided with an informed consent form, which they must sign. Both the Information Sheet and Informed Consent Form are available in S2 file.

The privacy and anonymity of research participants are rigorously protected, and all information or data collected for this research will only be disclosed with explicit consent or as mandated by law. Each participant’s name is securely stored in a password-protected database, associated solely with a study identification number, which is also utilized on all participant data sheets.

The findings of this study will be compiled into a report and distributed to PERKESO Rehabilitation Centre and Universiti Kebangsaan Malaysia. Upon completion and conclusion of the study, the raw materials will be kept in a locked file cabinet located in the Researcher’s office. Electronic data will be safeguarded and accessible only through a password known solely to the Researcher. Following five years from the completion of the study, all materials will be shredded and disposed of by the Researcher.

The trial results will be reported in accordance with the Consolidated Standards of Reporting Trials (CONSORT) guidelines. The manuscript detailing the trial will be submitted to a peer-reviewed international scientific journal for publication. Additionally, the findings may be presented at national conferences and scientific meetings.

## Results

This study was commenced on 15^th^ March 2022. Currently, the data collection process for protocol implementation is still ongoing.

## Discussion

Wrist fractures can produce residual disability and pain that may impact the functionality of a person. The application of robotic technology in facilitating upper limb movement and functional recovery training is extensive in the field of neurorehabilitation [42–51] but limited in the field of orthopaedic rehabilitation particularly in hand therapy practice. Given the current concepts of robotic intervention are focused on providing motor control/learning, practice-induced neuroplasticity, intensity, and task specific training, robotic intervention for orthopaedic hand and wrist disorders has been suggested [12]. Recent work has served as the introducing point to incorporate robotic devices in the rehabilitation program of traumatic wrist injuries. The study has shown that robotic-based rehabilitative approach is effective and comparable to the traditional therapy approach on individuals with wrist injuries [52].

This randomized controlled trial aims to investigate the effectiveness of combined conventional therapy and HAL-SJ robotic therapy in restoring the wrist functionality following the fractures as compared to the standard conventional therapy solely. Investigating the outcome of HAL-SJ among upper limb injured workers, especially those with wrist fractures shall provide further evidence on the effectiveness and feasibility of robotic interventions in the field of hand therapy and upper limb rehabilitation.

The effectiveness of the study protocol has been evaluated by examining the functional and impairment levels of the impacted hand. To compare wrist functionality and work readiness between control and intervention groups, the DASH and LASER outcome measures are employed. Additionally, the VAS, goniometric measurement, isometric strength assessment, Purdue Pegboard, and Box and Block Tests offer comparable data on impairment outcomes such as pain, strength, and hand dexterity for both groups.

Subsequent investigations ought to focus on assessing the effectiveness of HAL-SJ robotic therapy independently in restoring wrist functionality subsequent to traumatic wrist fractures, without integrating conventional therapy methods.

Here are several extra factors to contemplate -

- While a power analysis was conducted, it is important to note that the participants in this study might not entirely reflect the broader population of individuals with wrist fractures.
- The inclusion criteria did not rule out potential participants with associated fractures or traumas to other body parts.

## Supporting information

S1 File SPIRIT checklist. (PDF)

S2 File Study protocol. Study protocol with information sheet, consent form, demographic questionnaire, and data collection form.
(PDF)

S3 File Study protocol approved by ethics committee. Study protocol approved by the Research Ethics Committee of Universiti Kebangsaan Malaysia (JEP-2021–201).
(PDF)
