# Supplementary material for: Single Joint Hybrid Assistive Limb (HAL-SJ) robotic exoskeleton therapy in improving functional outcomes among workers with wrist fractures: Study protocol for a randomized controlled trial
Source: PLoS One. 2025 Apr 24;20(4):e0322191. doi: 10.1371/journal.pone.0322191 (PMC12021254; doi:10.1371/journal.pone.0322191)
Supplement: S2 File — Study protocol with information sheet, consent form, demographic questionnaire, and data collection form. (PDF) [file pone.0322191.s002.pdf]

Study Protocol

Week	Control Group & Intervention Group<br>(Conventional Therapy)	Intervention Group<br>(HAL-SJ Robotic Intervention)
1	Interventions to improve joint mobility and to reduce pain<br><br>Occupational Therapy:<br>Active and passive range of motion exercise of digits, elbow, and shoulder<br>Active range of motion exercise of wrist and forearm rotation<br>Tendon gliding exercise<br>Dexterity exercise<br><br>Physiotherapy:<br>Joint mobilization<br>Soft tissue mobilization<br>Retrograde massage<br>Heat/Cold modalities<br>Ultrasound<br>Electrical stimulation	HAL-SJ – Gentle Mode<br>i) Extension: 50 repetitions; 3 times<br>ii) Flexion: 50 repetitions; 3 times<br>iii) Supination: 50 repetitions; 3 times<br>iv) Pronation: 50 repetitions; 3 times<br><br>Parameters:<br>Assist Gain: 75-100<br>Assist Level: Normal<br>Flex/Ext Signal Balance: 100/100<br>Active/Relax Phase:<br>5seconds/5 seconds
2	Interventions to improve joint mobility and pain<br><br>PLUS<br><br>Occupational Therapy:<br>Functional activities training<br><br>Physiotherapy<br>Isometric strengthening exercises<br>Isotonic strengthening exercises	HAL-SJ – Gentle Mode<br>i) Extension: 50 repetitions; 3 times<br>ii) Flexion: 50 repetitions; 3 times<br>iii) Supination: 50 repetitions; 3 times<br>iv) Pronation: 50 repetitions; 3 times<br>Parameters:<br>Assist Gain: 50-75<br>Assist Level: Normal<br>Flex/Ext Signal Balance: 100% : 100%<br>Active/Relax Phase:<br>10 seconds/10 seconds

3	Interventions to improve joint mobility and pain<br><br>PLUS<br><br>Occupational Therapy:<br>Functional activities training<br><br>Physiotherapy:<br>Isometric strengthening exercises<br>Isotonic strengthening exercises<br>Progressive resisted exercises<br>Open kinetic chain activities<br>Closed kinetic chain activities	HAL-SJ – Gentle Mode<br>i) Extension: 50 repetitions; 3 times<br>ii) Flexion: 50 repetitions; 3 times<br>iii) Supination: 50 repetitions; 3 times<br>iv) Pronation: 50 repetitions; 3 times<br><br>Parameters:<br>Assist Gain: 25-50<br>Assist Level: Normal<br>Flex/Ext Signal Balance:<br>3 50% : 100%<br>4 100% : 50%<br>Active/Relax Phase:<br>10 seconds/10 seconds
4	Interventions to improve joint mobility and pain<br><br>PLUS<br><br>Occupational Therapy:<br>Work activity simulation<br>Work conditioning<br>Work hardening<br><br>Physiotherapy:<br>Isometric strengthening exercises<br>Isotonic strengthening exercises<br>Progressive resisted exercises<br>Open kinetic chain activities<br>Closed kinetic chain activities	HAL-SJ – Gentle Mode<br>i) Extension: 50 repetitions; 3 times<br>ii) Flexion: 50 repetitions; 3 times<br>iii) Supination: 50 repetitions; 3 times<br>iv) Pronation: 50 repetitions; 3 times<br><br>Parameters:<br>Assist Gain: 0-25<br>Assist Level: Normal<br>Flex/Ext Signal Balance:<br>i) 25% : 100%<br>ii) 100% : 25%<br>Active/Relax Phase:<br>10 seconds/10 seconds

Information Sheet
Randomized Controlled Trial

Research title:
The Effectiveness of Single Joint Hybrid Assistive Limb (HAL-SJ) Robotic Exoskeleton in Improving Functional Outcomes among Workers with Wrist Fractures: A Randomized Controlled Trial

Introduction
You are invited to participate in this research study because you have sustained a wrist fracture that had limited your performance in daily and work activities. Wrist fracture cases can take up to few months or even one to two years to achieve maximal functional improvement. The residual disability or pain, along with the long functional recovery period following wrist fracture may impact the socioeconomics of a person, family, and society significantly. Thus, a new intervention with the robotic exoskeleton will be implemented along with the standard hand therapy program to optimize the functional recovery process.

The details of the study will be described in this document. It is important for you to fully understand the purpose of this study and what it will involve. Kindly read through this information thoroughly for your consideration and decision to participate in this study. Ask the researcher if there is anything that is unclear or if you would like more information or clarification. Take time to decide whether or not you wish to take part.

Participation in this study is voluntary. If you agree to take part, then you will be asked to sign the “Informed Consent Form”. You will be given a copy of the consent form and this Information Sheet. If you do not agree to participate, you do not need to give any reason and nothing will be affected. If you decided to participate, you can still withdraw from the study at any time without any penalty. If you withdraw, any data collected from you up to your withdrawal will still be used for the study. Your refusal to participate or withdrawal will not affect any medical or health benefits to which you are otherwise entitled. The researcher may also remove you from the study for a variety of reason.

Purpose of study

The purpose of this study is to evaluate the effectiveness of single joint hybrid assistive limb (HAL-SJ) robotic exoskeleton in improving the functional outcomes among workers with wrist fractures. The application of robotic technology in facilitating upper limb movement and functional recovery training is extensive in the field of neurorehabilitation but limited in the field of orthopaedic rehabilitation particularly in hand therapy practice.

Robotic interventions had been proved to provide motor control/learning, practice-induced neuroplasticity, intensity, and task specific training for greater functional recovery. The upper limb HAL-SJ is a wearable movement support robot that detects bioelectrical signals on the skin surface and assists joint movements by controlling and operating an actuator placed outside the respective joint based on the frontier science “Cybernics”. Currently, there are vast of studies on the clinical applications of robotic interventions for the neurological population. However, the use of robotic interventions in orthopaedic rehabilitation especially for the wrist and hand remains inadequately explored. Therefore, this study aims to determine the effectiveness of robotic interventions in improving the functions as well as the recovery period following wrist fracture.

A total of 42 participants who sustained wrist fractures as similar to you will be recruited to participate in this study. Your participation in this study will only last for 4 weeks while the whole study will be implemented for 36 months.

What will the study involve?

If you agree to participate in the study, you will be asked to complete a series of questionnaires and complete a few measurements. You will then be randomly assigned to one of the treatment groups below by drawing from concealed envelopes. You have equal chance of being assigned to each of the groups.

Group 1:

a) Participants will undergo two conventional therapy sessions daily that include an occupational therapy session and a physiotherapy session on a 5-day/week routine basis.

Group 2:

a) Participants will undergo two conventional therapy sessions daily that include an occupational therapy session and a physiotherapy session on a 5-day/week routine basis.
b) Within two hours after the conventional therapy sessions, participants will undergo the HAL-SJ robotic therapy session conducted by the researcher at a separate treatment room on a 5-day/week routine basis.

Regardless of your group allocation, you will need to complete the following questionnaires and measurements administered by the therapist at the beginning of the hand therapy treatment (pre-intervention/initial assessment) and after 4 weeks of hand therapy treatment (post-intervention):

1) Functional performance by using Disabilities of the Arm, Shoulder and Hand (DASH) Outcome Measure
2) Work readiness by using LAM Assessment on Stages of Employment Readiness (LASER) Questionnaire
3) Pain level by using Visual Analogue Scale (VAS)
4) Physical performance of Active Range of Motion (AROM) of the hand and wrist by using goniometer
5) Physical performance of grip and pinch strength by using Jamar Dynamometer and B&L Engineering Pinch Gauge respectively
6) Physical performance of fine dexterity by using Purdue Pegboard Test (PPT)
7) Physical performance of gross manual dexterity by using Box and Block Test (BBT)

Post-intervention outcome measurements will be performed after the 20th therapy session, i.e., upon the completion of the 4-weeks program.

It is important that you answer all of the questions asked by the therapist honestly and completely. You must inform the therapist if your condition or circumstances change during the study or if you make any changes to any of your current treatments.

After the trial, you will continue receiving the necessary therapy sessions as usual. Whether you complete the study or withdraw early, the best alternatives for your future treatment will be discussed with the treating team.

Risks and benefits:

Single Joint Hybrid Assistive Limb (HAL-SJ) robotic exoskeleton is certified by ISO 13485:2016 (certificate number: 1757.181211) and EC (registration number: DD 601417310001) for rehabilitation and physical therapy usage.

The potential risks of participating in the Single Joint Hybrid Assistive Limb (HAL-SJ) robotic exoskeleton session may include:

a) Skin allergy or reddening of area where electrode is affixed. However, the reddening will disappear shortly after the electrode was taken off
b) Abrasion of areas that contact the device e.g. cuffs and straps
c) Muscle and joint soreness (due to post exercise effect)

The researcher of this study, who is a certified HAL-SJ operator accredited by Cyberdyne Inc. will take necessary precaution and careful supervision throughout the entire Single Joint Hybrid Assistive Limb (HAL-SJ) robotic exoskeleton session to minimise these potential risks. You are advised to communicate and inform the researcher immediately if you have any concern of the above risks.

By participating in this study, you will understand the performance of your physical and psychological functions before and after the therapy program. If you are being allocated to the intervention group that receives an extra Single Joint Hybrid Assistive Limb (HAL-SJ) robotic exoskeleton session, your performance of physical and psychological functions will facilitate our understanding regarding the therapeutic effects of Single Joint Hybrid Assistive Limb (HAL-SJ) robotic exoskeleton in wrist fracture rehabilitation.

Do you have to take part?

Participation in this study is voluntary. If you agree to take part, then you will be asked to sign the "Informed Consent Form". You will be given a copy of the consent form and this Information Sheet. Throughout your participation, you may also refuse to answer any questions that you do not want to answer. If you are not agreeing to participate, you do not need to give any reason and nothing will be affected. If you decided to participate, you can still withdraw from the study at any time without any penalty. If you withdraw, any data collected from you up to your withdrawal will still be used for the study. Your refusal to participate or withdrawal will not affect any medical or health benefits to which you are otherwise entitled.

Data and confidentiality

All your information obtained in this study will be kept and handled in a confidential manner, in accordance with applicable laws and/or regulations. The data from this study will be made into a report which may be published. Once the data have been gathered and the research completed, the raw materials will be kept in the locked file cabinet in the office of the researcher. Electronic data will be securely kept using password. Access to the data is only by the researcher. After 5 years, the material will be shredded and discarded. The data will be reported in a collective manner with no reference to an individual.

Payment and compensation

This study is self-funded by the researcher himself. No external funding or grant is received. Therefore, no payment will be given to you for participating in this study. If you are allocated into Group 2 (intervention group), you will not be charged for the additional Single Joint Hybrid Assistive Limb (HAL-SJ) robotic exoskeleton sessions received.

Can this study or my participation be terminated early?

The researcher may stop the study or your participation at any time due to the concerns of your safety. If the study is stopped early for any reason, you will be informed and arrangements will be made for your future care. You will have the opportunity to see your own measurement results upon completion of this study.

Who should I call if I have questions?

If you have any questions about the study or if you think you have a study related injury and you want information about treatment, please contact the following researchers:

Dr. Chai Siaw Chui
Main Supervisor
Occupational Therapy Programme
Faculty of Health Sciences
Universiti Kebangsaan Malaysia
Telephone No.: 03-92897047
Email: sc.chai@ukm.edu.my

Mr. Tan Eng Wah
Master’s Candidate
Occupational Therapy Programme
Faculty of Health Sciences
Universiti Kebangsaan Malaysia
Telephone No.: 012-6358531
Email: tanew9911@gmail.my

INFORMED CONSENT FORM

Title of Study: The Effectiveness of Single Joint Hybrid Assistive Limb (HAL-SJ) Robotic Exoskeleton in Improving Functional Outcomes among Workers with Wrist Fractures: A Randomized Controlled Trial

By signing below I confirm the following:

- I have been given oral and written information for the above study and have read and understood the information given.
- I have had sufficient time to consider participation in the study and have had the opportunity to ask questions and all my questions have been answered satisfactorily.
- I understand that my participation is voluntary and I can withdraw from the study at any time without giving a reason and this will not affect my future treatment. I am not taking part in any other research study at this time. I understand the risks and benefits, and I freely give my informed consent to participate under the conditions stated. I understand that I must follow the researcher instructions related to my participation in the study.
- All personal details will be treated as CONFIDENTIAL.
- I will receive a copy of this subject information/informed consent form signed and dated to bring home.

Subject:

Signature:	I/C number:
Name:	Date:

Investigator conducting informed consent:

Signature:	I/C number:
Name:	Date:

Impartial witness:

Signature:	I/C number:
Name:	Date:

Sociodemographic Questionnaire

ID: _______________ Date: _____________

Please complete the following items by providing the answers in the space provided:	
Age	
Gender	Male / Female
Race	Malay/ Chinese/ Indian/ Others (please specify)
Marital Status	Married/ Single/ Divorced
Occupation	
Educational Level	UPSR/ PMR/ SPM/ STPM/ Certificate/ Diploma/ Bachelor's Degree/ Master's Degree/ PhD<br>No formal education
Hand Dominance	Right/ Left/ Ambidextrous
Diagnosis	
Date of Injury	
Date of Referral	
History of Present Injury	
Mechanism of injury	
Medical/ Surgical Management	Conservative/ Operative/ Conservative & Operative
Types of Medical/ Surgical Interventions	
Date of Surgery	
Types of Wrist Fracture	Extra-articular/ Partial Articular/ Intra-Articular
Employment Status	Employed/ Unemployed
Monthly Salary	
Medical Leave	Yes / No
SOCSO Compensation Scheme	Employment Injury / Invalidity

Data Collection Form

1) Disabilities of the Arm, Shoulder and Hand (DASH) Outcome Measure

	Initial Assessment<br>Date:	Reassessment<br>Date:
DASH Symptom Score		

2) LAM Assessment on Stages of Employment Readiness (LASER) Questionnaire

	Initial Assessment<br>Date:	Reassessment<br>Date:
LASER Score		

3) Visual Analogue Scale (VAS)

	Initial Assessment<br>Date:	Reassessment<br>Date:
VAS Score		

4) Grip Strength

	Initial Assessment<br>Date:	Reassessment<br>Date:
1st Trial		
2nd Trial		
3rd Trial		
Average		

5) Lateral Pinch Strength

	Initial Assessment<br>Date:	Reassessment<br>Date:
1st Trial		
2nd Trial		
3rd Trial		
Average		

6) Tripod Pinch Strength

	Initial Assessment<br>Date:	Reassessment<br>Date:
1st Trial		
2nd Trial		
3rd Trial		
Average		

7) Tip-to-tip Pinch Strength

	Initial Assessment<br>Date:	Reassessment<br>Date:
1st Trial		
2nd Trial		
3rd Trial		
Average		

8) Active Range of Motion – Forearm

	Initial Assessment<br>Date:	Reassessment<br>Date:
Supination		
Pronation		

9) Active Range of Motion – Wrist

	Initial Assessment<br>Date:	Reassessment<br>Date:
Extension		
Flexion		
Radial Deviation		
Ulnar Deviation		

10) Active Range of Motion – Hand

		Initial Assessment<br>Date:	Reassessment<br>Date:
Thumb	MCPJ Flexion		
	IPJ Flexion		
Index Finger	MCPJ Flexion		
	PIPJ Flexion		
	DIPJ Flexion		
Middle Finger	MCPJ Flexion		
	PIPJ Flexion		
	DIPJ Flexion		
Ring Finger	MCPJ Flexion		
	PIPJ Flexion		
	DIPJ Flexion		
Little Finger	MCPJ Flexion		
	PIPJ Flexion		
	DIPJ Flexion		

11) Purdue Pegboard Test

Initial Assessment Date:

	1st Trial	2nd Trial	3rd Trial	Average
Right Hand				
Left Hand				
Both Hand				
R + L + B				
Assembly				

Reassessment Date:

	1st Trial	2nd Trial	3rd Trial	Average
Right Hand				
Left Hand				
Both Hand				
R + L + B				
Assembly				

12) Box and Block Test

Initial Assessment Date:

	Right Hand	Left Hand
Number of Blocks Transferred in 60 Seconds		

Reassessment Date:

	Right Hand	Left Hand
Number of Blocks Transferred in 60 Seconds		
