# Supplementary material for: Single Joint Hybrid Assistive Limb (HAL-SJ) robotic exoskeleton therapy in improving functional outcomes among workers with wrist fractures: Study protocol for a randomized controlled trial
Source: PLoS One. 2025 Apr 24;20(4):e0322191. doi: 10.1371/journal.pone.0322191 (PMC12021254; doi:10.1371/journal.pone.0322191)
Supplement: S3 File — Study protocol approved by the Research Ethics Committee of Universiti Kebangsaan Malaysia (JEP-2021–201). (PDF) [file pone.0322191.s003.pdf]

THE EFFECTIVENESS OF SINGLE JOINT HYBRID ASSISTIVE LIMB (HAL-SJ) ROBOTIC EXOSKELETON THERAPY IN IMPROVING FUNCTIONAL OUTCOMES AMONG WORKERS WITH WRIST FRACTURES: A RANDOMIZED CONTROLLED TRIAL

PREPARED BY:

TAN ENG WAH (P101608)

SUPERVISOR:

DR. CHAI SIAW CHUI

CO-SUPERVISORS:

PROF. DR. YOSHIYUKI SANKAI

DR. NOR AFIFI RAZAOB@RAZAB

DR. MASAHIRO SHINGU

1.0 INTRODUCTION

The primary goal of occupational therapy is to enable people to participate in the activities of daily living (ADLs) including the ability to work (Desiron et al. 2011).

Work-related hand injuries contributed to 24.9% of all industrial accidents seen at the emergency departments and orthopaedic clinics in Malaysia (Al-Husuny et al. 2012). Approximately 98.6 % of workers with traumatic hand injuries returned to work after a median absence of 45.5 days (Oberfeld et al. 2015).

Wrist joint is a joint with high activity frequencies and high functional requirements (Weimer et al. 1999). The wrist is divided into three major joint articulations, namely the distal radioulnar joint, the radiocarpal joint and the midcarpal joint (Berge 1996; Kawamura & Chung 2007). It consists of eight carpal bones interposed between the forearm (distal radius and ulna) and the bases of the metacarpals (Berger 1996; Calfee et al. 2010). The carpal bones are categorized as a proximal row and distal row based on their kinematics during wrist motions (Berger1996; Kijima & Viegas, 2009; Calfee et al. 2010). The proximal row consists of the scaphoid, lunate, triquetrum, and pisiform while the distal row consists of the trapezium, trapezoid, capitate, and hamate (Berger, 1996; Kawamura & Chung, 2007; Kijima & Viegas, 2009; Calfee et al. 2010). The distal radioulnar joint (DRUJ) consists of the ulna head, the sigmoid notch region of the distal radius, the DRUJ capsule, and the triangular fibrocartilage complex (TFCC) (Berger, 1996; Kawamura & Chung, 2007). The wrist and distal radioulnar joint combine to form an essential joint capable of providing motions with six degrees of freedom in the cardinal planes. These include flexion, extension, radial deviation, ulnar deviation, pronation, and supination that result in circumduction (Calfee et al. 2010).

Based on the wrist anatomical structures, wrist fractures may include fractures that affect the distal ends of the radius, ulna, and carpus. A distal radius fracture, commonly known as a wrist fracture, is defined by the involvement of the metaphysis of the distal radius. The fracture may or may not involve the radiocarpal joint, distal radioulnar joint, and/or the distal ulna (Mauck & Swigler 2018). Traditionally, distal

radius fractures have been described using eponyms, e.g. Colles, Smith, Barton, and Chauffeur. During the last half of the twentieth century, several classification systems for distal radius fractures have emerged which include the AO Foundation/Orthopaedic Trauma Association (AO/OTA) system (Kleinlugtenbelt et al. 2017; Mulders et al. 2017; Wæver et al. 2018), the Frykman system (Meena et al. 2014; Kleinlugtenbelt et al. 2017; Mulders et al. 2017; Wæver et al. 2018), the Melone system (Meena et al. 2014; Mulders et al. 2017), the Fernandez system (Meena et al. 2014; Kleinlugtenbelt et al. 2017; Mulders et al. 2017), and the Older system (Wæver et al. 2018). According to Wæver et al. (2018), the AO/OTA classification system seems to be most reliable for routine use while the study by Mulders et al. (2017) found high consensus among both surgeons and residents for defining the AO/OTA classification as the preferred classification system.

According to the fracture and dislocation classification compendium by the AO Foundation/Orthopaedic Trauma Association (AO/OTA) (2018), the distal end segment fractures for both the radius and ulna bones are classified into three types, namely extra articular fracture, partial articular fracture, and complete articular fracture. The distal end segment radius and ulna fractures are further classified into nine and three groups respectively. The nine groups of distal end segment radius fractures include radial styloid avulsion fracture, simple fracture, wedge or multifragmentary fracture, sagittal fracture, dorsal rim (Barton's) fracture, volar rim (reverse Barton's, Goyrand Smith II's) fracture, simple articular and metaphyseal fracture, metaphyseal multifragmentary fracture, articular multifragmentary fracture, and simple or multifragmentary metaphyseal fracture. Meanwhile, the three groups of distal end segment ulna fractures include extraarticular, styloid process fracture, simple fracture, and multifragmentary fracture.

Incidence of distal radius fracture is increasing worldwide in which one sixth of the fractures treated in emergency rooms appears to be related to distal radius (Mitchlovitz et al. 2001; MacIntyre & Dewan 2016). Majority of patients had some degrees of disability one year after distal radius fracture with 16% had moderate to very severe disability and were not working (Moore & Leonardi-Bee 2008). Patients who have high self-reported pain/disability and occupational demand at baseline have an

average of 9.2 weeks of work loss following distal radius fracture (MacDermid et al. 2007). Hand injury costs had brought cumulative economy impact not only to patients, but their family, employer, and society (Dias & Garcia-Elias 2006). As the incidence of distal radius fractures rises, the short and long term costs become apparent (Nellan et al. 2012).

Stiffness and pain (Egol et al. 2014) are common complications of distal radius fractures. Loss of range of motion (ROM) is typically due to periarticular connective tissue changes after fractures, joint injuries, and immobilization (Mitchlovitz et al. 2004). Decreased wrist flexion and/or extension after trauma or surgery can be a challenging problem (McGracth et al. 2008). The ultimate goal of restoring motion is to reduce impairments and enhance functional performance for ADLs, work, and leisure.

The rehabilitation program following distal radius fractures involves three phases: immobilization phase, mobilization phase, and strengthening phase. The fundamental goals throughout these three phases include effective edema control and pain management as well as ROM, strength, and functional restorations (Mitchlovitz et al. 2001). The most frequently used therapeutic interventions following distal radius fractures include compressive wrap with retrograde massage, heat/cold modalities, joint mobilization, electrical stimulation, continuous passive motion (CPM), soft-tissue mobilization, ultrasound, splinting, ROM and strengthening exercises, and functional training (Mitchlovitz et al. 2001). The commonly used nonsurgical interventions for ROM restoration after fracture, fracture/dislocation, joint injury, or other soft tissue injuries include home exercise program and clinic based program such as splinting and casting, joint mobilization, CPM, and therapeutic exercises (Mitchlovitz et al. 2004).

Improper rehabilitation program after wrist injury may significantly impair the overall function of the upper extremity (Garcia-Elias & Folgar 2006). Both injury and patient factors can impact the management and outcomes after distal radius fracture. Although optimal strength, ROM, and function are achievable within 3-6 months of conservative or surgical management (MacDermid et al. 2007), some patients require longer duration to complete the rehabilitative phase. According to Moore and

Leornardi-Bee (2008), there are 11% of patients endure residual moderate to very severe pain one year following distal radius fracture with 16% still experiencing ongoing disability. This signifies that there is a significant association between patient characteristics and the level of pain and disability among the distal radius fracture population.

With respect to the employability issues of the workers with wrist fractures, Lam et al. (2010) had advocated that matching treatment to the patient's stage of employment readiness appears to be a more effective approach to service provision. A timely and goals oriented treatment-matching approach in the work program will deliver better outcomes in term of cost effectiveness, employment and service quality. The Lam Assessment on Stages of Employment Readiness (LASER) has been used in the treatment-matching endeavour by assessing the patient's stages of employment readiness based on Prochaska's State of Change theory that consists of three stages, namely the Pre-Contemplation, Contemplation, and Action stages (Chan et al. 2006; Lam et al. 2010).

The application of robotic technology in facilitating upper limb movement and functional recovery training is extensive in the field of neurorehabilitation (Riener et al. 2005; Riener 2007; Wagner et al. 2011; Lo & Xie 2012; Ren et al. 2013; Maciejasz et al. 2014; Borboni et al. 2016; Zhang et al. 2017; Hsieh et al. 2018; Ferreira et al. 2018) but limited in the field of orthopaedic rehabilitation particularly in hand therapy practice. Given the current concepts of robotic intervention are focused on providing motor control/learning, practice-induced neuroplasticity, intensity, and task specific training, Hakim et al. (2017) have suggested the application of robotic intervention in orthopaedic rehabilitation with upper extremity disorders, especially those related to hand and wrist.

There are various types of robotic devices that can be used for upper limb rehabilitation, subject to their respective mechanical designs (i.e. end-effector-based or exoskeleton-based) and types of assistance provided (i.e. active, passive, haptic or coaching) (Maciejasz et al., 2014). The Single Joint Hybrid Assistive Limb (HAL-SJ) is one of the robotic devices that can be used for upper limb rehabilitation. Hybrid

Assistive Limb (HAL) is a cyborg type robot that can expand, augment, and support physical capability in order to enhance and upgrade human capabilities based on the frontier science "Cybernics". Cybernics is a new domain of interdisciplinary research centered on cybernetics, mechatronics, and informatics that integrates neuroscience, robotics, systems engineering, information technology, "kansei" engineering, ergonomics, physiology, social science, law, ethics, management, economics, etc. (Sankai 2010).

The HAL is a hybrid control system that consists of a 'Cybernic Voluntary Control (Bio-Cybernic Control)' and 'Cybernic Autonomous Control (Cybernic Robot Control)'. The cybernic control system can provide suitable physical support to wearers with various conditions including both healthy persons and physically challenged persons by using the two types of algorithms as complementary controls. The Cybernic Voluntary Control provides physical supports/actions according to the operator's voluntary intention caused by the bioelectrical signals including muscle activity. The power units of HAL generate power assist torque by amplifying the wearer's own joint torque estimated from his/her bioelectrical signals, and the support motions are consequently controlled. Bioelectrical signals, including myoelectricity, are useful and reliable information to estimate a human's motion intentions because the signals are measured just before corresponding visible muscle activities. Thus, the wearer receives physical support directly by an unconscious interface using the bioelectrical signals. In contrary, the Cybernic Autonomous Control autonomously provides a desired functional motion generated according to the wearer's body constitution, conditions, and purposes of motion support (Sankai 2010).

The upper limb HAL-SJ is a wearable movement support robot that detects bioelectrical signals on the skin surface and assists joint movements by controlling and operating an actuator placed outside the respective joint (Otsuka et al. 2011; Kubota et al. 2018). Upper limb rehabilitation using the elbow joint HAL-SJ robotic therapy has been applied to promote elbow functions among neurological patients. Functional imaging study had shown cortical neuroplasticity activity associated with elbow movements among post-stroke patients following 20 sessions of elbow joint HAL-SJ intervention for the paretic arm in two weeks period (Saita et al. 2016). In addition,

studies had proved the safety and feasibility of the upper limb HAL-SJ application for the upper limb rehabilitation of quadriplegia patients (Kubota et al. 2018; Shimizu et al. 2017). The upper limb HAL-SJ is also proven safe and effective to be used for the rehabilitation following traumatic brachial plexus injury (Kubota et al. 2017). A newly developed wrist joint attachment to the HAL-SJ is currently available and is capable of assisting voluntary articulatory movements of the wrist joint including wrist flexion and extension as well as forearm supination and pronation.

Robotic technologies have been developed for motor rehabilitation and such robots have shown favourable results when compared with equivalent doses of usual clinical therapy (Lo et al. 2010; Novak 2018). Compared to manual therapy, robotic intervention for rehabilitation has the potential for a greater impact on impairment due to easy deployment, its applicability across of a wide range of motor impairment, its high measurement reliability, and the capacity to deliver high dosage and high intensity training protocols for a longer duration, irrespective of the skills, and fatigue level of the therapist (Huang & Krakauer 2009). Based on the understanding that the amount and intensity of exercise and the quality of motor learning are positively associated with rehabilitation outcome as aforementioned and the fact that HAL-SJ is a newly developed robotic intervention device that shares similarities on the therapeutic features provided by other robotic devices, the application of the HAL-SJ is hypothesized to provide similar positive outcome for wrist fractures. Investigating the outcome of HAL-SJ among upper limb injured workers, especially those with wrist fractures shall provide further evidence on the effectiveness and feasibility of robotic interventions in the field of hand therapy and upper limb rehabilitation.

2.0 PROBLEM STATEMENT

Most patients recovered from wrist fracture, particularly the distal radius fracture have some residual disability or pain. MacDermid et al. (2003) found that at one year following distal radius fracture, of 129 patients, there were still 46% of them experienced certain extent of difficulty with usual activities, ranging from very severe to mild. Although majority of recovery following a distal radius fracture occurred within six-months after conventional hand therapy regime, most of the patients can be expected

to still experience minimal pain and disability. Distal radius fracture cases can take up to few months and one or two years to achieve maximal functional improvement (Michlovitz et al., 2001). The residual disability or pain, along with the long functional recovery period following distal radius fracture may impact the socioeconomics of a person, family, and society significantly. With the advancement in rehabilitation technology, robotic intervention had shown positive outcomes in neurological rehabilitation programme for the past decades. However, there is a lacking of published evidence about the feasibility and effectiveness of robotic intervention in orthopaedic rehabilitation programme, especially in the field of hand therapy.

2.1 RESEARCH JUSTIFICATION

Upper limb robotic interventions have shown promising results and positive evidence in functional recovery of the neurological population. Robotic interventions are capable to provide highly repetitive, intensive, and task-specific training required for functional restoration based on current concepts of motor learning, practiced-induce neuroplasticity and muscle plasticity. Currently, there are vast of studies on the clinical applications of robotic interventions for neurological rehabilitation. However, the use of robotic interventions in orthopaedic rehabilitation especially for the wrist and hand remains inadequately explored. This study aims to determine the effectiveness of robotic interventions in improving the functions as well as the recovery period following distal radius fracture.

2.2 RESEARCH QUESTION

Does a combined conventional therapy with Single Joint Hybrid Assistive Limb (HAL-SJ) robotic therapy provide greater effectiveness in improving functional level, physical performance and work readiness among workers with wrist fractures compared to conventional therapy alone?

2.3 HYPOTHESIS

Hypothesis 1: A 4-week combined conventional therapy and Single Joint Hybrid Assistive Limb (HAL-SJ) robotic therapy provides greater functional level improvement among workers with wrist fractures as compared to those receiving conventional therapy alone during the same intervention period as measured by the Disabilities of the Arm, Shoulder, and Hand (DASH) Outcome Measure.

Hypothesis 2: A 4-week combined conventional therapy and Single Joint Hybrid Assistive Limb (HAL-SJ) robotic therapy provides greater improvement in term of pain reduction among workers with wrist fractures as compared to those who only receive standard conventional therapy in the same intervention period as measured by the Visual Analogue Scale (VAS).

Hypothesis 3: A 4-week combined conventional therapy and Single Joint Hybrid Assistive Limb (HAL-SJ) robotic therapy provides greater improvement in term of wrist and fingers range of motion among workers with wrist fractures as compared to those who only receive standard conventional therapy in the same intervention period as measured by the goniometer.

Hypothesis 4: A 4-week combined conventional therapy and Single Joint Hybrid Assistive Limb (HAL-SJ) robotic therapy provides greater improvement in term of grip strength among workers with wrist fractures as compared to those who only receive standard conventional therapy in the same intervention period as measured by the Jamar dynamometer.

Hypothesis 5: A 4-week combined conventional therapy and Single Joint Hybrid Assistive Limb (HAL-SJ) robotic therapy provides greater improvement in term of pinch strength among workers with wrist fractures as compared to those who only receive standard conventional therapy in the same intervention period as measured by the B&L Engineering pinch gauge.

Hypothesis 6: A 4-week combined conventional therapy and Single Joint Hybrid Assistive Limb (HAL-SJ) robotic therapy provides greater improvement in term of fine dexterity of hand function among workers with wrist fractures as compared to those who only receive standard conventional therapy in the same intervention period as measured by the Purdue Pegboard Test.

Hypothesis 7: A 4-week combined conventional therapy and Single Joint Hybrid Assistive Limb (HAL-SJ) robotic therapy provides greater improvement in term of gross dexterity of hand function among workers with wrist fractures as compared to those who only receive standard conventional therapy in the same intervention period as measured by the Box and Block Test.

Hypothesis 8: A 4-week combined conventional therapy and Single Joint Hybrid Assistive Limb (HAL-SJ) robotic therapy provides greater improvement in work readiness among workers with wrist fractures as compared to those receiving conventional therapy alone during the same intervention period as measured by the Lam Assessment of Stages of Employment Readiness (LASER).

2.4 RESEARCH OBJECTIVE

2.4.1 General Objective

The objective of this research is to determine the effectiveness of Single Joint Hybrid Assistive Limb (HAL-SJ) robotic therapy in improving functional level, physical performance, and work readiness among workers with wrist fractures after 4 weeks of intervention.

2.4.2 Specific Objective

a. Specific objective 1:

To determine the effectiveness of Single Joint Hybrid Assistive Limb (HAL-SJ) robotic therapy on functional level among workers with wrist fracture after 4 weeks of intervention.

c. Specific objective 2:

To determine the effectiveness of Single Joint Hybrid Assistive Limb (HAL-SJ) robotic therapy on physical performance in terms of pain level, wrist and fingers range of motion, grip and pinch strength as well as hand dexterity among workers with wrist fracture after 4 weeks of intervention.

d. Specific objective 3:

To determine the effectiveness of Single Joint Hybrid Assistive Limb (HAL-SJ) robotic therapy on work readiness among workers with wrist fracture after 4 weeks of intervention.

2.5 CONCEPTUAL FRAMEWORK

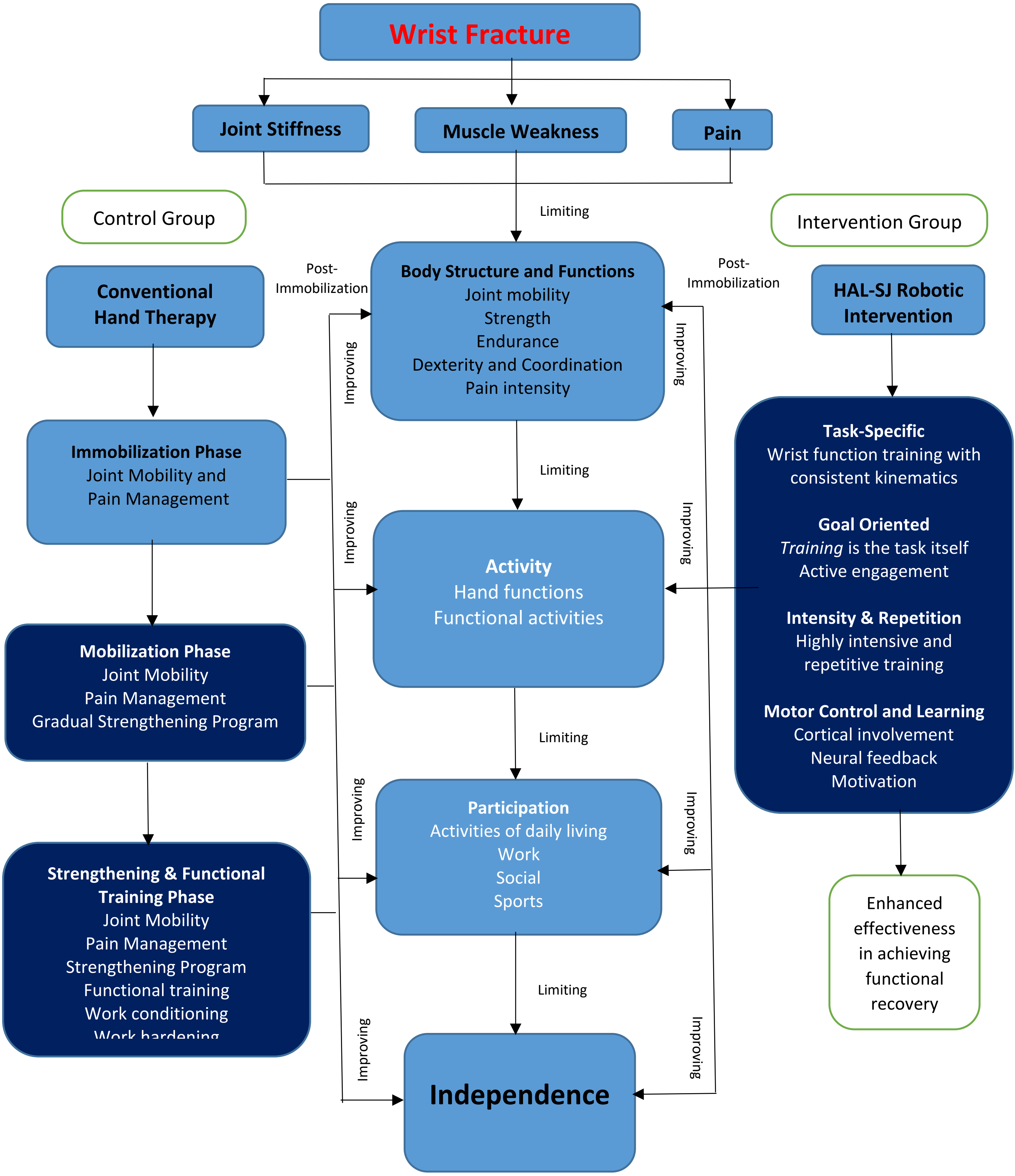

3.0 METHODOLOGY

3.1 STUDY DESIGN

This research will use a randomized controlled trial (RCT) design where the effects of the study treatment (intervention) are compared with those of a control treatment after the patients were being randomly assigned to the two groups i.e., the control group and the intervention group.

3.2 STUDY POPULATION

The target participants are Social Security Organisation (SOCSO) insured workers with wrist fractures that match both the inclusion and exclusion criteria and receiving rehabilitation management at PERKESO Rehabilitation Centre. The PERKESO Rehabilitation Centre is selected in this study because the researcher is working at this facility which provides convenience for him to carry out the research and intervention for the participants. In addition, PERKESO Rehabilitation Centre is a subsidiary body under SOCSO that appears to be one of the largest rehabilitation facilities in Malaysia. It plays a vital role in providing free comprehensive and intensive rehabilitation program to all injured workers covered under the SOCSO scheme.

3.3 SAMPLE SIZE

The sample size of each hypothesis is calculated by using the G* power analysis. Given that this is a novel study and there is no reference from previous study, sample size was calculated using ANOVA repeated measures, within-between interaction with Cohen-f effect size at 0.25 for a medium effect, alpha error at 0.05 and 80% power. Result showed that a total of 34 participants will be needed. By adding 10% dropout rate, the required sample size became 38 participants.

INCLUSION AND EXCLUSION CRITERIA

3.4.1 Inclusion criteria

a. SOCSO insured Malaysian workers who are on medical leave; have not returned to work; and receiving rehabilitation program at PERKESO Rehabilitation Centre.
b. Wrist fracture involving individual or combined distal radius, distal ulna, distal radioulnar, radiocarpal joint
c. Extraarticular fracture, partial articular fracture, and/or complete articular fracture

3.4.2 Exclusion criteria

a. Aged below 18 years old or above 60 years old (based on SOCSO scheme)
b. Associated peripheral nerve injuries, neuropathy or/and tendon injuries to the affected upper limb
c. Associated neurological conditions related to the central nervous system
d. Cognitive impairment or psychiatric condition
e. Severe and uncontrolled medical conditions e.g. cardiovascular disorders, respiratory disorders, cancer
f. Cardiac pacemaker
g. Skin disorders e.g. eczema, psoriasis, open would etc. of the affected forearm and hand that prevent electrode placements
h. Severe contracture of the affected wrist joint
i. Forearm size that is not fit to wear the Single Joint Hybrid Assistive Limb (HAL-SJ) robotic exoskeleton

3.5 RESEARCH PROCEDURE

a. This research will involve the researcher, Therapist A, Therapist B, Therapist C, the intervention group and the control group.
b. The researcher is a certified HAL-SJ operator accredited by Cyberdyne Inc. and is competent to conduct the robotic intervention for the intervention group (Appendix A).

c. Therapist A will be an experienced occupational therapist in the field of hand therapy. She/ He will be given the role as the baseline and outcome assessor as well as conducting the conventional therapy interventions that include ROM exercises, tendon gliding exercise, dexterity exercise, retrograde massage, splinting, progressive functional strengthening, functional activity simulation, work conditioning, and work hardening.
d. Therapist B will be an experienced physiotherapist who is responsible to conduct the conventional therapy interventions that include heat/cold modalities, therapeutic ultrasound, electrical stimulation, joint mobilization, soft tissue mobilization, and strengthening exercises.
e. All patients with wrist fracture who are referred to PERKESO Rehabilitation Centre will be categorized through the admission database. Participants will be selected based on the inclusion and exclusion criteria by the Disability Management Department which manages the patient admission procedure. The selected participants will be informed about the study and only be included in the study after giving their informed consent.
f. The participants will then be randomly assigned to the control and intervention group respectively. Simple randomization with allocation concealment is used to prevent the researcher from influencing which participants are assigned to the intervention group.
g. An independent Therapist C who is not assessing or treating the participants will be given an equal numbers of random allocation cards with designated alphabet of "C" (Control group) and "I" (Intervention group). Therapist C will then insert each allocation card into an opaque envelope. Each envelope that contains a random allocation card with either "C" or "I" notation will be sealed and enclosed with glue. Therapist C will give the corresponding envelope to each enrolled participant after he/she has completed all the baseline assessment. The selected envelope will be opened by the participant immediately in the presence of Therapist C. Therapist C subsequently records the allocated group of either "C" or "I" for the respective participant on the excel sheet prepared by the researcher. The information regarding participant group allocation will only be known by the researcher and Therapist C. Both the Therapist A and Therapist B will not be informed about the group allocation of each participant.

h. Both participants from the control and intervention groups will be receiving conventional therapy that includes splinting, scar management, edema management, physical agent modalities, e.g. hot/cold pack, ultrasound, Transcutaneous Electrical Nerve Stimulation (T.E.N.S.), Neuromuscular Electrical Stimulation (N.M.E.S.), passive/active stretching, joint mobilization, strengthening exercises, and hand function training.

i. Each participant from both the control and intervention groups will receive two conventional therapy sessions daily that include an occupational therapy session and a physiotherapy session on a 5-day/week routine basis. Each occupational therapy and physiotherapy session will last for 90 minutes respectively. In addition to the conventional therapy, the intervention group will receive a 60-minutes HAL-SJ robotic therapy session conducted by the researcher daily on a 5-day/week routine basis within two hours after the conventional therapy sessions.

j. All participants will be receiving this 5-day/week therapy sessions for a duration of four weeks. Reassessment of therapeutic outcome for both control and intervention groups will be conducted by the Therapist A upon completion of the therapy program after 4 weeks.

3.5.1 PROTOCOL FOR CONVENTIONAL THERAPY AND SINGLE JOINT HAL-SJ ROBOTIC INTERVENTION

Week	Control Group & Intervention Group<br>(Conventional Therapy)	Intervention Group<br>(HAL-SJ Robotic Intervention)
1	Interventions to improve joint mobility and to reduce pain:<br><br>Occupational Therapy:<br>Active and passive range of motion exercise of digits, elbow, and shoulder<br>Active range of motion exercise of wrist and forearm rotation	HAL-SJ – Gentle Mode<br>i) Extension: 50 repetitions; 3 sets<br>ii) Flexion: 50 repetitions; 3 set<br>iii) Supination: 50 repetitions; 3 sets<br>iv) Pronation: 50 repetitions; 3 sets<br><br>Parameters:<br>Assist Gain: 75-100<br>Assist Level: x1

	Tendon gliding exercise<br>Dexterity exercise<br><br>Physiotherapy:<br>Joint mobilization<br>Soft tissue mobilization<br>Retrograde massage<br>Heat/Cold modalities<br>Ultrasound<br>Electrical stimulation	Flexion/Extension Signal Balance:<br>100%/100%<br>Active/Relax Phase:<br>10 seconds/10 seconds
2	Interventions to improve joint mobility and pain<br><br>PLUS<br>Occupational Therapy:<br>Functional activities training<br><br>Physiotherapy<br>Isometric strengthening exercises<br>Isotonic strengthening exercises	HAL-SJ – Gentle Mode<br>i) Extension: 50 repetitions; 3 sets<br>ii) Flexion: 50 repetitions; 3 sets<br>iii) Supination: 50 repetitions; 3 sets<br>iv) Pronation: 50 repetitions; 3 sets<br><br>Parameters:<br>Assist Gain: 50-75<br>Assist Level: x1<br><br>Flexion/Extension Signal Balance:<br>100% : 100%<br>Active/Relax Phase:<br>10 seconds/10 seconds
3	Interventions to improve joint mobility and pain<br><br>PLUS<br>Occupational Therapy:<br>Functional activities training<br><br>Physiotherapy:<br>Isometric strengthening exercises	HAL-SJ – Gentle Mode<br>i) Extension: 50 repetitions; 3 sets<br>ii) Flexion: 50 repetitions; 3 sets<br>iii) Supination: 50 repetitions; 3 sets<br>iv) Pronation: 50 repetitions; 3 sets<br><br>Parameters:<br>Assist Gain: 25-50<br>Assist Level: x1

	Isotonic strengthening exercises<br>Progressive resisted exercises<br>Open kinetic chain activities<br>Closed kinetic chain activities	Flexion/Extension Signal Balance:<br>i) 50% : 100%<br>ii) 100% : 50%<br>Active/Relax Phase:<br>10 seconds/10 seconds
4	Interventions to improve joint mobility and pain<br><br>PLUS<br>Occupational Therapy:<br>Work activity simulation<br>Work conditioning<br>Work hardening<br><br>Physiotherapy:<br>Isometric strengthening exercises<br>Isotonic strengthening exercises<br>Progressive resisted exercises<br>Open kinetic chain activities<br>Closed kinetic chain activities	HAL-SJ – Gentle Mode<br>i) Extension: 50 repetitions; 3 sets<br>ii) Flexion: 50 repetitions; 3 sets<br>iii) Supination: 50 repetitions; 3 sets<br>iv) Pronation: 50 repetitions; 3 sets<br><br>Parameters:<br>Assist Gain: 0-25<br>Assist Level: x1<br>Flexion/Ext Signal Balance:<br>i) 25% : 100%<br>ii) 100% : 25%<br>Active/Relax Phase:<br>10 seconds/10 seconds

3.6 STUDY INSTRUMENTS

3.6.1 Single Joint Hybrid Assistive Limb (SJ-HAL) Robotic Exoskeleton

Single Joint Hybrid Assistive Limb (HAL-SJ) robotic exoskeleton is certified by ISO 13485:2016 (certificate number: 1757.181211) and EC (registration number: DD 601417310001) for rehabilitation and physical therapy usage. The upper limb HAL-SJ is a wearable movement support robot that detects bioelectrical signals on the skin surface and assists joint movements by controlling and operating an actuator placed outside the respective joint. The power units of HAL generate power assist torque by amplifying the wearer’s own joint torque estimated from his/her bioelectrical signals, and the support

motions are consequently controlled. It is a safe device that can be used for rehabilitation intervention as approved by the FDA, EC and MDA. It may cause several minor problems: (a) skin allergy or reddening of area where electrode is affixed, however, the reddening should disappear shortly after the electrode was taken off; (b) abrasion of areas that contact the device e.g. cuffs and straps; and (c) muscle and joint soreness (due to post exercise effect). The researcher of this study, who is a certified HAL-SJ operator accredited by Cyberdyne Inc. will take necessary precaution and careful supervision throughout the entire HAL-SJ robotic exoskeleton session to minimise these problems. The medical and healthcare teams of the facility (PERKESO Rehabilitation Centre) including the doctors, medical assistants, and nurses will be consulted immediately if the problems are serious enough to warrant medical treatment. The HAL-SJ robotic exoskeleton that will be used is shown as Figures 1 and 2.

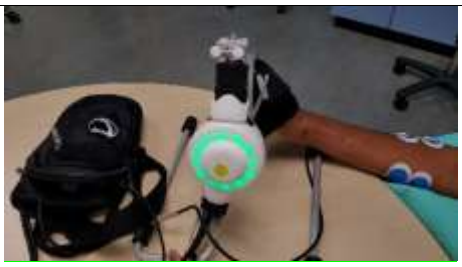	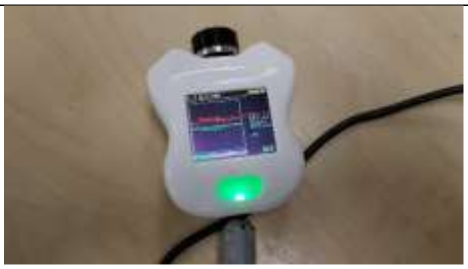
Figure 1	Figure 2

Given outcome measures commonly used for wrist fractures include wrist ROM measurement, grip/pinch strength measurement (with dynamometer and pinch gauge), pain measurement (Visual Analogue Scale), and the Disabilities of the Arm, Shoulder and Hand (DASH) Outcome Measure (Weinstock-Zlotnick & Metha 2016), all these outcome measures will be used in this study. In addition, both Purgue Pegboard Test and Box and Block Test will be used to evaluate the functional performance in term of hand dexterity while the readiness to work will be evaluated by using the LAM Assessment on Stages of Employment Readiness (LASER) Questionnaire.

3.6.2 Sociodemographic Questionnaire

This self-developed questionnaire will be used to collect participant’s personal information, including age, gender, occupation, hand dominance, history of present injury including date of injury, mechanism of injury, medical/surgical

management and date of surgery, employment status, and compensation scheme. The Sociodemographic Questionnaire is shown as in Appendix B.

3.6.3 Disabilities of the Arm, Shoulder and Hand (DASH) Outcome Measure

The Disabilities of the Arm, Shoulder and Hand (DASH) Outcome Measure is an extensively researched evaluative and discriminative region-specific patient-rated outcome measure used by many clinicians and researchers in the field of hand therapy. This instrument was first developed by the American Academy of Orthopedic Surgeons, the Council of the Musculoskeletal Speciality Societies, and the Institute for Work and Health (IWH), Toronto (Ontario), and published in 1996 (Hudak et al. 1996; Klerk et al. 2018). The DASH was developed to be a measure of physical function and symptoms for any or multiple musculoskeletal conditions of the upper limb (Kennedy & Beaton 2017). The DASH has multiple language versions including the Malay (Al-Husuny et al. 2011) and Simplified Chinese (Chen et al. 2015). There is extensive research evidence of its psychometric properties (Klerk et al. 2018). The DASH is a 30-item questionnaire that evaluates symptoms and physical function (at the level of disability), with a five response option for each item. The total score of DASH is calculated using the provided formula with higher score indicates greater disability (MacDermid 2000).

DASH score formula:

Total score $= \left(\left[\frac{sum\ of\ n\ responses}{n}\right] - 1\right) \times 25$

Evidence was provided of the validity, test-retest reliability, and responsiveness of the DASH. The DASH is useful across the whole upper extremity with its demonstrated validity and responsiveness in both proximal and distal disorders (Beaton et al. 2001). According to study by Beaton et al. (2001), the DASH outcome measure exceeded recommended standards for test-retest reliability for both individual-and group-level interpretation of the scores (Intraclass correlation coefficient, ICC=0.96). Convergent construct validity was shown by demonstrating moderate to high correlations with other markers of disability and symptoms (Pearson Product Moment Correlation Coefficient = 0.68–0. 92 while Spearman correlations=0.69–0.92). Discriminative validity was confirmed by showing differences between the DASH scores of patients who were working

and those who were not (26.8 vs. 50.7, t=-7.51, p<0.0001) as well as patients who were functioning and the scores of those who were not (23.6 vs. 47.1, t= -5.81, p<0.0001). The DASH is responsive to the different types of change before and after treatment of the target conditions (Standardized Response Mean, SRM=0.74-0.80) and in those patients whose ability to function had improved (SRM=0.92-1.40). The Malay version of DASH has internal consistency, Cronbach's α 0.99 and test-retest reliability, ICC=0.94) (Al-Husuny et al. 2011). The DASH Outcome Measure that will be used (English, Malay, and Chinese versions) are attached as Appendices C, D and E.

3.6.4 LAM Assessment on Stages of Employment Readiness (LASER) Questionnaire

The Lam Assessment on Stages of Employment Readiness (LASER) was developed to measure one's psychological readiness to return to work after an extended period of unemployment due to disability (Lam et al. 2010). LASER contains 14 items corresponding to Prochaska's stages of change model which describe behaviours in the pre-contemplation (6 statements), contemplation (4 statements) and action stages (4 statements). The workers are asked to rate each item on a five-point Likert Scale with "1" indicating strongly disagree to "5" indicating strongly agree. The scores are then added and allocated under different sub-scores representing the corresponding stages as forming a continuous measure. The highest sub-score will represent a subject's tendency towards the corresponding stage. Subjects at the pre-contemplation (PC) stage do not see unemployment as a problem, and are often not interested in working, or believe that they cannot work. Subjects at the contemplation (C) stage begin to consider the pros- and cons- of working, but they have not yet participated in any related action such as job searching. Subjects at the action (A) stage have decided to work by engaging in job seeking behaviours and removing many of the barriers related to returning to work (Chan et al. 2006). The correlations between the factors were −0.50 (between PC and C), −0.56 (between PC and A), and 0.74 (between C and A), and the correlations between the three pairs of error terms were 0.34, 0.33, and 0.27. The Cronbach's alphas (a measure of internal consistency) for the Pre-contemplation and Contemplation scales are 0.77 and the Action scale is 0.82 (Lam et al. 2010). The LASER Questionnaire has been

translated to both Chinese and Malay languages. Both translated versions of LASER Questionnaires have shown good test retest reliability. The intraclass correlation coefficient (ICC) for the Chinese version ranged from 0.55 to 0.79 (Chan et al. 2006) while the ICC for the Malay version ranged between 0.707 and 0.917 (Chandran & Jamaludin 2009). The LASER Questionnaire (English, Malay, and Chinese versions) that will be used are attached as Appendices F, G, and H.

3.6.5 Visual Analogue Scale (VAS)

The Visual Analogue Scale (VAS) is a reliable and valid instrument commonly used for measurement of pain intensity in rehabilitation (Crossley et al. 2004). It is a single-item instrument that measures the whole construct at once. It consists of a 100mm horizontal line anchored with two opposite labels, i.e. no pain and severe pain; patients mark a score on the scale using a vertical line. Because VAS is easy to use, it is applicable to a variety of practice and research settings (Boonstra et al. 2008). The VAS that will be used are attached as Appendix I.

3.6.6 Goniometer

Range of motion measurement will be done by using the goniometer. Active and passive ROM of the wrist and fingers of the fractured wrist will be assessed (Adams et al. 2003; Bland et al. 2008; Goldhahn et al. 2014; Waljee et al. 2016). Active ROM of the wrist joint, including wrist flexion, wrist extension, wrist radial deviation, wrist ulnar deviation will be assessed first, followed by AROM of the forearm rotation i.e. supination and pronation. Following AROM, passive ROM will be moved to end range in order to assess end feel. Passive ROM begins with the wrist flexion, wrist extension, wrist radial deviation, and wrist ulnar deviation. Following wrist motion, passive forearm rotation, i.e., supination and pronation will be tested to assess movement ability of proximal and distal radioulnar joints (Porretto-Loehrke et al. 2016). Active and passive ROM of the fingers' metacarpophalangeal joint (MCPJ), proximal interphalangeal joint (PIPJ), and distal interphalangeal joint (DIPJ) will be

assessed as well. The ROM measurements will be recorded using Clinical Data Record Sheet as shown in Appendix J.

3.6.7 Hand dynamometer and Pinch Gauge

Grip strength will be assessed with a Jamar hand dynamometer. Each participant's grip strength will be measured 3 times. Handle position 2 will be used as it is a recommended position for measuring grip strength by the American Society of Hand Therapists (Fess 1992). Meanwhile, lateral pinch strength, tripod pinch strength and tip-to-tip pinch strength will be measured by using the B&L Engineering Pinch Gauge (Mathiowetz et al. 2000). The grip and pinch strength measurements will be recorded using Clinical Data Record Sheet as shown in Appendix J.

3.6.8 Purdue Pegboard Test (PPT)

The Purdue Pegboard Test (PPT) is a standardized assessment consisting of four different subtests to determine user's level of dexterity. It is composed of a board with pins, collars, and washers. In the first three subtests, the subject has to place the maximum numbers of pins into holes in the pegboard with the dominant hand, the non-dominant hand, and then with both hands simultaneously within 30 seconds. In the fourth subtest the subject uses alternate hands in order to make assemblies consisting of pins, collars, and washers, in 60 seconds. A fifth score is given by summarizing the first three sub-tests (Shahar et al. 1998). The PPT was identified as one of the top three assessments of hand dexterity for health care professionals, due to its relatively higher reliability and validity and fewer confounding variables, such as age, gender, and handedness (Lindstrom-Hazel & Veenstra 2015). The Purdue Pegboard Test results will be recorded using Clinical Data Record Sheet as shown in Appendix J.

3.6.8 Box and Block Test (BBT)

The Box and Block Test (BBT) is a functional outcome measure that measures the gross manual dexterity and is commonly used across multiple clinical populations due to its benefits of ease and speed of implementation (Mathiowetz et al. 1985; Kontson et al. 2017). In the BBT, the subject is required to transfer

one hundred and fifty 2.5cm3 wooden blocks that are placed in many different orientations on the side of the partition with the testing hand as fast as possible to another partition in one minute. A subject’s score is equal to the number of these blocks transported over a 15.2-cm tall partition in one minute (Mathiowetz et al. 1985; Konston et al. 2017). The Box and Block Test had shown a high inter-rater and test-retest reliability with interclass correlations coefficients of 0.85 to 0.97 (Desrosiers et al. 1994; Platz et al. 2005; Canny et al. 2009). Mathiowetz et al. 1985 had published the normative data for the B&B Test for adults on the basis of 628 people without disabilities between ages 20 and 70+ years. The Box and Block Test results will be recorded using Clinical Data Record Sheet as shown in Appendix J.

3.7 ETHICAL CONSIDERATION

Application of ethics for this research will be sent to the *Jawatankuasa Etika Penyelidikan Universiti Kebangsaan Malaysia* (JEPUKM) and the Scientific Committee of PERKESO Rehabilitation Centre. The study will commence after obtaining approval from both authority parties. Respective clinicians and staff dealing with hand injuries’ patients will be informed about the study. Participation is on voluntary basis and participants have rights to withdraw from the study at any stage if they wish to do so without the exercise of any pressure or coercion. The participants will be given an information sheet about the study. Informed consent form will be given and signed by participants that agreed to participate in the study. The Information Sheet and Informed Consent Form are shown as in Appendix K. The protection of the privacy and anonymity of research participants are ensured and all information or data used for this research will only be released with consent or if required by law. The study will be registered at the Australian New Zealand Clinical Trials Registry (ANZCTR).

The ethical application for research and intervention implementation at PERKESO Rehabilitation Centre had been approved by the Scientific Committee of

PERKESO Rehabilitation Centre on the 18th November 2020 as shown with the attached Appendix L.

3.8 STATISTICAL ANALYSIS

All data will be analysed using the IBM® SPSS® Statistics version 28. The study will use both descriptive and inferential statistics to analyse the data. Descriptive analysis for categorical data including gender, marital status, hand dominance, types of medical management and intervention, type of wrist fractures, educational level, employment status, and type of SOCSO compensation scheme will be expressed in frequency, percentage or/and proportion. Descriptive statistics for continuous data including age, DASH, pain, grip and pinch strength, wrist and finger ROM, hand dexterity, and work readiness scores will be expressed in mean, standard deviation, maximum, and minimum if the data is normally distributed or in median and inter-quartile range if the data in not normally distributed. The Shapiro-Wilks test will be used to test the normality of the continuous data.

The statistical analysis for each hypothesis will be tested using mixed model ANOVA by evaluating the mean differences in treatment outcomes between the groups at baseline and after 4 weeks of intervention.

Intention-to-treat (ITT) analysis will be used to analyze the difference between groups. ITT analysis, which aims to minimize bias and maintain random allocation of study participants (Gupta 2011), is suitable to be used when there are shedding participants. Its key idea is to analyse the group to which the participant belongs in that initial allocation, regardless of whether they completed the study of received the intended intervention.

3.9 RESEARCH FLOW CHART

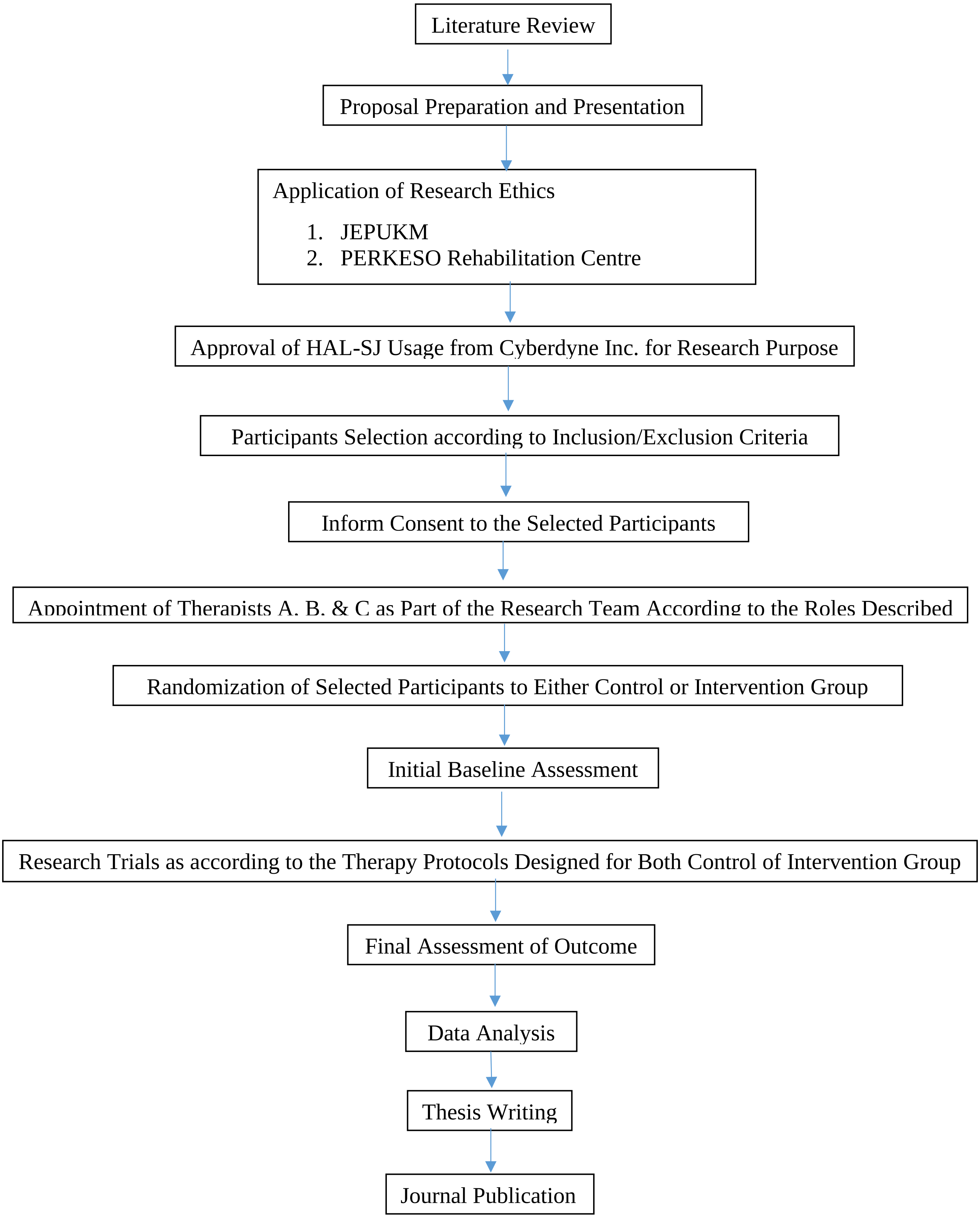

3.10 GANTT CHART

	2020											
	Jan	Feb	Mar	Apr	May	Jun	Jul	Aug	Sep	Oct	Nov	Dec
Proposal Preparation	√	√										
Proposal Presentation	√	√										
Application of Ethics Approval											√	√
Participants Recruitment and Selection												
Intervention Implementation												
Data Collection												
Data Analysis												
Chapter 1	√	√	√	√	√	√	√	√	√	√	√	√
Chapter 2	√	√	√	√	√	√	√	√	√	√	√	√
Chapter 3							√	√	√	√	√	√
Chapter 4												
Chapter 5												
Chapter 6												

	2021											
	Jan	Feb	Mar	Apr	May	Jun	Jul	Aug	Sep	Oct	Nov	Dec
Proposal Preparation												
Proposal Presentation												
Application of Ethics Approval	√	√										
Participants Recruitment and Selection			√	√	√	√	√	√	√	√	√	√
Intervention Implementation			√	√	√	√	√	√	√	√	√	√
Data Collection			√	√	√	√	√	√	√	√	√	√
Data Analysis												
Chapter 1	√	√	√	√	√	√	√	√	√	√	√	√
Chapter 2	√	√	√	√	√	√	√	√	√	√	√	√
Chapter 3	√	√	√	√	√	√	√	√	√	√	√	√
Chapter 4												
Chapter 5												
Chapter 6												

	2022											
	Jan	Feb	Mar	Apr	May	Jun	Jul	Aug	Sep	Oct	Nov	Dec
Proposal Preparation												
Proposal Presentation												
Application of Ethics Approval												
Participants Recruitment and Selection	√	√	√	√	√	√	√	√	√	√	√	√
Intervention Implementation	√	√	√	√	√	√	√	√	√	√	√	√
Data Collection	√	√	√	√	√	√	√	√	√	√	√	√
Data Analysis									√	√	√	√
Chapter 1	√	√	√	√	√	√	√	√	√	√	√	√
Chapter 2	√	√	√	√	√	√	√	√	√	√	√	√
Chapter 3	√	√	√	√	√	√	√	√	√	√	√	√
Chapter 4												
Chapter 5												
Chapter 6												

	2023											
	Jan	Feb	Mar	Apr	May	Jun	Jul	Aug	Sep	Oct	Nov	Dec
Proposal Preparation												
Proposal Presentation												
Application of Ethics Approval												
Participants Recruitment and Selection	√	√	√	√	√	√	√	√	√	√	√	√
Intervention Implementation	√	√	√	√	√	√	√	√	√	√	√	√
Data Collection	√	√	√	√	√	√	√	√	√	√	√	√
Data Analysis	√	√	√	√	√	√	√	√	√	√	√	√
Chapter 1	√	√	√	√	√	√	√	√	√	√	√	√
Chapter 2	√	√	√	√	√	√	√	√	√	√	√	√
Chapter 3	√	√	√	√	√	√	√	√	√	√	√	√
Chapter 4	√	√	√	√	√	√	√	√	√	√	√	√
Chapter 5	√	√	√	√	√	√	√	√	√	√	√	√
Chapter 6	√	√	√	√	√	√	√	√	√	√	√	√

	2024											
	Jan	Feb	Mar	Apr	May	Jun	Jul	Aug	Sep	Oct	Nov	Dec
Proposal Preparation												
Proposal Presentation												
Application of Ethics Approval												
Participants Recruitment and Selection	√	√	√	√	√	√	√	√	√	√	√	√
Intervention Implementation	√	√	√	√	√	√	√	√	√	√	√	√
Data Collection	√	√	√	√	√	√	√	√	√	√	√	√
Data Analysis	√	√	√	√	√	√	√	√	√	√	√	√
Chapter 1	√	√	√	√	√	√	√	√	√	√	√	√
Chapter 2	√	√	√	√	√	√	√	√	√	√	√	√
Chapter 3	√	√	√	√	√	√	√	√	√	√	√	√
Chapter 4	√	√	√	√	√	√	√	√	√	√	√	√
Chapter 5	√	√	√	√	√	√	√	√	√	√	√	√
Chapter 6	√	√	√	√	√	√	√	√	√	√	√	√

	2025											
	Jan	Feb	Mar	Apr	May	Jun	Jul	Aug	Sep	Oct	Nov	Dec
Proposal Preparation												
Proposal Presentation												
Application of Ethics Approval												
Participants Recruitment and Selection	√	√	√	√	√	√						
Intervention Implementation	√	√	√	√	√	√						
Data Collection	√	√	√	√	√	√						
Data Analysis	√	√	√	√	√	√						
Chapter 1	√	√	√	√	√	√						
Chapter 2	√	√	√	√	√	√						
Chapter 3	√	√	√	√	√	√						
Chapter 4	√	√	√	√	√	√						
Chapter 5	√	√	√	√	√	√						
Chapter 6	√	√	√	√	√	√						

REFERENCES

1. Adams, B. D., Grosland, N. M., Murphy, D. M. & McCullough, M. 2003. Impact of impaired wrist motion on hand and upper-extremity performance. *Journal of Hand Surgery*. 28 (6): 898-903.

2. Alhusuny, A., Rampal, L., Manohar, A., Adon, M. Y. & Ahamd, A. A. 2012. Work-related hand injuries: type, location, cause, mechanism and severity in a tertiary hospital. *Malaysian Journal of Medicine and Health Sciences.* 8(2):41-49.

3. Alhusuny, A., Rampal, L., Arumugam, M., Rahman, H. & Zakaria, J. 2011, November 14-15. Validation of a Malay version of disability of arm, shoulder and hand questionnaire. 3rd Asia Pacific Conference on Public Health, Kuala Lumpur, Malaysia.

4. Beaton, D. E., Katz, J. N., Fossel, A. H., Wright, J. G., Tarasuk, V. & Bombardier, C. 2001. Measuring the whole or the parts? Validity, reliability and responsiveness of the disabilities of the arm, shoulder and hand outcome measure in different regions of the upper extremity. *Journal of Hand Therapy*. 14(2):128-46.

5. Berger, R. A. 1996. The anatomy and basic biomechanics of the wrist joint. *Journal of Hand Therapy*. 9(2), 84-93.

6. Bland, M., Beebe, J. A. & Hardwick, D. D. 2008. Restricted active range of motion at the elbow, forearm, wrist, or fingers decreases hand function. *Journal of Hand Therapy*.21:268–75.

7. Boonstra, A. M., Preuper, H. R. S., Reneman, M. F., Posthumus, J. B. & Stewart, R. E. 2008. Reliability and validity of the visual analogue scale for disability in patients with chronic musculoskeletal pain. *International Journal of Rehabilitation Research.* 31:165–169.

8. Borboni, A., Villafañe, J. H., Mullè, C., Valdes, K., Faglia, R., Taveggia, G. & Negrini,S. 2016. Robot-assisted rehabilitation of hand paralysis after stroke reduces wrist edema and pain: a prospective clinical trial. *Journal of Manipulative and Physiological Therapeutics.*

9. Calfee, R. P., Berger, R., Beredjiklian, P. K., Engles, D. R., Greenberg, J. A., Isaacs, J. E., McDaniel, C., Rozental, T. D. & Pate, O. 2010. Fractures and dislocations: Wrist. In Hammert, W. C., Calfee, R. P., Bozentka, D. J. & Boyer, M. I. (Eds.), *ASSH manual of hand surgery* (pp. 216-254). Philadelphia: Wolters Kluwer Health/Lippincott Williams & Wilkins.

10. Campbell, D. A. & Wilkinson, T. C. 2011. (ii) Wrist fractures. *Orthopaedics and Trauma.* 25(5), 324-335.

11. Canny, M. L., Thompson, J. M. & Wheeler, M. J. 2009. Reliability of the box and block test of manual dexterity for use with patients with fibromyalgia. *The American Journal of Occupational Therapy.* 63(4): 506-510.

12. Chan, H., Li-Tsang, C. W., Lam, C. S., Hui, K. L. & Bard, C. 2006. Validation of Lam assessment of employment readiness (CLASER) for Chinese injured workers. *Journal of Occupational Rehabilitation.* 16:697–705.

13. Chandran, V. J. & Jamaludin, N. E. 2009. Validation of Lam assessment of stages of employment readiness for Malay spoken workers. [Unpublished bachelor’s degree dissertation]. Universiti Teknoligi MARA.

14. Chen, H., Ji, X., Zhang, W., Zhang, Y., Zhang, L. & Tang, P. 2015. Validation of the simplified Chinese (Mainland) version of the disability of the arm, shoulder, and hand questionnaire (DASH-CHNPLAGH). *Journal of Orthopaedic Surgery and Research.* 10:76.

15. Crossley, K. M., Bennell, K. L., Cowan, S. M. & Green, S. 2004. Analysis of outcome measures for persons with patellofemoral pain: which are reliable and valid? *Archive of Physical Medicine and Rehabilitation.* 85:815–822.

16. de Klert, S., Buchanan, H. & Jerosch-Herold, C. 2018. The validity and clinical utility of the disabilities of the arm shoulder and hand questionnaire for hand injuries in developing country contexts: A systematic review. *Journal of Hand Therapy.* 31: 80-90.

17. Désiron, H. A. M., de Rijk, A., Van Hoof, E. & Donceel, P. 2011. Occupational therapy and return to work: a systemic literature review. *BMC Public Health.* 11:615.

18. Desrosiers, J., Bravo, G., Hebert, R., Dutil, E. & Mercier, L. 1994. Validation of the Box and Block Test as a measure of dexterity of elderly people: Reliability, validity, and norms studies. *Archives of Physical Medicine and Rehabilitation*. 75(7): 751-755.

19. Desrosiers, J., Rochette, A., Hebert, R. & Bravo, G. 1997. The Minnesota manual dexterity test: reliability, validity and reference values studies with healthy elderly people. *Canadian Journal of Occupational Therapy.* 4(5): 271-276.

20. Dias, J. J. & Garcia-Elias, M. 2006. Hand injury costs. *Injury, International Journal of the Care of the Injured.* 37: 1071—1077.

21. Egol, K. A., Karia, R., Zingman, A., Lee, S. & Paksima, N. 2014. Hand stiffness following distal radius fractures. Who gets it and is it a functional problem? *Bulletin of the Hospital for Joint Disease.* 72(4):288-93.

22. Ferreira, F. M. R. M., Chaves, M. E. A., Oliveira, V. C., Petten, A. M. V. N. V & Vimieiro, C. B. S. 2018. Effectiveness of robot therapy on body function and

structure in people with limited upper limb function: A systematic review and meta-analysis. *PLoS ONE.* 13(7).

23. Garcia-Elias, M & Folgar, M. A. V. 2006. The management of wrist injuries: An international perspective. *Injury, International Journal of the Care of the Injured.* 37: 1049—1056.

24. Goldhahn, J., Beaton, D., Ladd, A., Macdermid, J. & Hoang-Kim, A. 2014. Recommendation for measuring clinical outcome in distal radius fractures: A core set of domains for standardized reporting in clinical practice and research. *Archives of Orthopaedic and Trauma Surgery.* 134:197–205.

25. Fess. E. E. 1992. Grip strength. In: Casanova, J. S. (Ed.), *Clinical Assessment Recommendations, ed 2* (pp. 41–45). Chicago: American Society of Hand Therapists.

26. Hakim, R. M., Tunis, B. G. & Ross, M. D. 2017. Rehabilitation robotics for the upper extremity: review with new directions for orthopaedic disorders. *Disability & Rehabilitation: Assistive Technology.* 12 (8): 765-771.

27. Hazel, D.K.L & Veenstra, N.V.V. 2015. Examining the Purdue pegboard test for occupational therapy practice. *Open Journal of Occupational Therapy.* 3(3).

28. Hsieh, Y. W., Lin, K. C., Wu, C. Y., Shih, T. Y., Ming-wei Li, M. W. & Chen, C. L. 2018. Comparison of proximal versus distal upper-limb robotic rehabilitation on motor performance after stroke: a cluster controlled trial. *Scientific Reports.* 8:2091

29. Huang, V. S & Krakauer, J. W. 2009. Robotic neurorehabilitation: a computational motor learning perspective. *Journal of NeuroEngineering and Rehabilitation.* 2009:6.

30. Hudak, P. L., Amadio, P. C., Bombardier, C. & Upper Extremity Collaborative Group (UECG). 1996. Development of an upper extremity outcome measure: The DASH (Disabilities of the Arm, Shoulder, and Hand). *American Journal of Industrial Medicine.* 29:602-608.

31. Lindstrom-Hazel, D. K. & Veenstra, N.V. 2015. Examining the Purdue pegboard test for occupational therapy practice. *Open Journal of Occupational Therapy*. 3:3.

32. Kawamura, K. & Chung, K. C. 2007. Management of wrist injuries. *Plastic and Reconstructive Surgery*. 120(5): 73e-89e.

33. Kendall, J. M. 2003. Designing a research project: randomised controlled trials and their principles. *Emergency Medicine Journal*. 20: 164-168.

34. Kennedy, C. A. & Beaton, D. E. 2017. A user's survey of the clinical application and content validity of the DASH (Disabilities of the Arm, Shoulder and Hand) outcome measure. *Journal of Hand Therapy.* 30(1):30-40.

35. Kijima, Y. & Viegas, S. F. 2009. Wrist anatomy and biomechanics. *The Journal of Hand Surgery*. 34(8): 1555-1563.

36. Kim, J. & Shin, W. 2014. How to do random allocation (randomization)? *Clinics in Orthopedic Surgery.* 6(1): 103-109.

37. Kleinlugtenbelt, Y. V., Groen, S. R., Ham, S. J., Kloen, P., Haverlag, R., Simons, M. P, Scholtes, V. A. B., Bhandari, M., Goslings, J. C. & Poolman, W. R. (2017). Classification systems for distal radius fractures. Does the reliability improve using additional computed tomography? *Acta Orthop*. 88(6): 681–687.

38. Konston, K., Marcus, I., Myklebust, B. & Civillico, E. 2017. Targeted box and blocks test: normative data and comparison to standard tests. *PLoS ONE*. 12(5): e0177965.

39. Kubota, S., Hara, Y., Shimizu, Y., Kadone, H., Kubo, T., Marushima, A., Ueno, T., Kawamoto, H., Koda, M., Matsumura, A., Hada, Y., Sankai, Y. & Yamazaki, M. 2017. A newly developed upper limb single-joint HAL in a patient with elbow flexion reconstruction after traumatic brachial plexus injury: a case report. *Interdisciplinary Neurosurgery: Advanced Techniques and Case Management.* 10: 66-68.

40. Kubota, S., Abe, T., Koda, M., Kadone, H., Shimizu, Y., Mataki, Y., Noguchi, H., Fujii, K., Marushima, A., Funayama, T., Kawamoto, H., Hada, Y., Sankai, Y. & Yamazaki, M. 2018. Application of a newly developed upper limb single-joint hybrid assistive limb for postoperative C5 paralysis: An initial case report indicating its safety and feasibility. *Journal of Clinical Neuroscience.* 50: 268–271.

41. Lam, C. S., Wiley, A. H., Siu, A. & Emmett, J. 2008. Assessing readiness to work from a stages of change perspective: Implications for return to work. *Work*. 37: 321-329.

42. Lo, A. C., Guarino, P. D., Richards, L. G., Haselkorn, J. K., Wittenberg, G. F., Federman, D. G., Ringer, R. J., Wagner, T. H., Krebs, H. I., Volpe, B. T., Bever, C. T., Bravata, D. M., Duncan, P. W., Corn, B. H., Maffucci, A. D., Nadeau, S. E., Conroy, S. S., Poell, J. M., Huang, G. D. & Peduzzi, P. 2010. Robot-assisted therapy for long-term upper-limb impairment after stroke. *New England Journal of Medicine.* 362(19):1772–83.

43. Lo, H. S. & Xie, S. Q. 2012. Exoskeleton robots for upper-limb rehabilitation: State of the art and future prospects. *Medical Engineering & Physics.* 34: 261–268.

44. MacDermid, J. C. & Bellamy, N. 2000. Responsiveness of the short form-36, disability of the arm, shoulder, and hand questionnaire, patient-rated wrist evaluation, and physical impairment measurements in evaluating recovery after a distal radius fracture. *The Journal of Hand Surgery*. 25A(2): 330-340.

45. MacDermid, J. C., Roth, J. H. & Richards, R. S. 2003. Pain and disability reported in the year following a distal radius fracture: A cohort study. *BMC Musculoskeletal Disorders*. 4:24.

46. MacDermid, J. C., Roth, J. H. & McMurtry, R. 2007. Predictors of time lost from work following a distal radius fracture. *Journal of Occupational Rehabilitation.* 17:47–62.

47. Maciejasz, P., Eschweiler, J., Gerlach-Hahn, K., Jansen-Troy, A. & Leonhardt, S. 2014. A survey on robotic devices for upper limb rehabilitation. *Journal of NeuroEngineering and Rehabilitation.* 11:3.

48. MacIntyre, N. J. & Dewan, N. 2016. Epidemiology of distal radius fractures and factors predicting risk and prognosis. *Journal of Hand Therapy.* 29: 136-145.

49. Mathiowetz, V., Volland, G., Kashman, N. & Weber, K. 1985. Adult norms for the Box and Block Test of manual dexterity. *American Journal of Occupational Therapy*. 39(6):386–91.

50. Mathiowetz, V., Vizenor, L. & Melander, D. 2000. Comparison of baseline instruments to the Jamar dynamometer and the B&L engineering pinch gauge. *The Occupational Therapy Journal of Research*. 20(3):147-162.

51. Mauck, B. M. & Swigler, C. W. 2018. Evidence-based review of distal radius fractures. *Orthop. Clin. North Am*. 49(2): 211-222.

52. McGrath, M. S., Ulrich, S. D., Bonutti, P. M., Smith, J. M., Seyler, T. M. & Mont, M. A. 2008. Evaluation of static progressive stretch for the treatment of wrist stiffness. *Journal of Hand Surgery.* 33(9), 1498-1504.

53. Meena, S., Sharma, P., Sambharia, A. K. & Dawar, A. 2014. Fractures of distal radius: An overview. *Journal of Family Medicine and Primary Care.* 3(4): 325-332.

54. Mehta, S., MacDermid, J. & Tremblay, M. The implications of chronic pain models for rehabilitation of distal radius fracture. *Journal of Hand Therapy*. 16(1): 2-11.

55. Michlovitz, S. L., LaStayo, P. C., Alzner, S. & Watson, E. 2001. Distal radius fractures: Therapy practice patterns. *Journal of Hand Therapy.* 14:249-257.

56. Michlovitz, S. L., Harris, B. & Watkins, M. P. 2004. Therapy interventions for improving joint range of motion: a systematic review. *Journal of Hand Therapy*. 17:118–30.

57. Moore, C. M. & Leonardi-Bee, J. 2008. The prevalence of pain and disability one year post fracture of the distal radius in a UK population: A cross sectional survey. *BMC Musculoskeletal Disorders.* 9:129.

58. Mulders, M. A. M., Rikli, D., Goslings, J. C. & Schep, N. W. L. 2017. Classification and treatment of distal radius fractures: a survey among orthopaedic trauma surgeons and residents. *Eur J Trauma Emerg Surg*. 43:239–248.

59. Nellans, K. W., Kolwaski, E. & Chung, K. C. 2012. The Epidemiology of distal radius fractures. *Hand Clinics.* 28(2): 113–125.

60. Novak, D. 2018. Promoting motivation during robot-assisted rehabilitation. *Rehabilitation Robotics.* 11: 149-156.

61. Oberfeld, E., Zwahlen, M. & Vögelin, E. 2015. Return to Work after Traumatic Hand Injuries: Medical, Personal and Work-related Factors. *Handchir Mikrochir Plast Chir.* 47: 44–57.

62. Otsuka, T., Kawaguchi, K., Kawamoto, H. & Sankai, Y. 2011. Development of Upper-limb type HAL and reaching movement for meal-assistance. *IEEE. International Conference on Robotics and Biomimetics*

63. Platz, T., Pinkowski, C., van Wijck, F., Kim, I., de Bella, P. & Johnson, G. 2005. Reliability and validity of arm function assessment with standardized guidelines for the FugI-Meyer Test, Action Research Arm Test and Box and Block Test: a multicentre study. *Clinical Rehabilitation*. 19: 404-411.

64. Porretto-Loehrke, A., Schuh, C. & Szekeres, M. 2016. Clinical manual assessment of the wrist. *Journal of Hand Therapy.* 29(2):123-35.

65. Ren,Y., Kang, S. H. , Hyung-Soon Park, H. S., Yi-Ning Wu, Y. N., & Zhang L. Q. 2013. Developing a multi-joint upper limb exoskeleton robot for diagnosis, therapy, and outcome evaluation in neurorehabilitation. *IEEE Transactions on Neural Systems and Rehabilitation Engineering.* 21 (3).

66. Riener, R., Nef, T. & Colombo, G. 2005. Robot-aided neurorehabilitation of the upper extremities. *Medical & Biological Engineering & Computing.* 43: 2-10.

67. Riner, R. 2007. Robot-aided rehabilitation of neural function in the upper extremities. *Acta Neurochirurgica Supplement.* 97(1): 465–471.

68. Shahar, R. B., Kizony, R. & Nota, A. 1998. Validity of the Purdue pegboard test in assessing patients after traumatic hand injury. *Work.* 11: 315-320.

69. Schweighofer, N., Choi, Y. & Winstein, C. & Gordon, J. 2012. Task-oriented rehabilitation robotics. *American Journal of Physical Medicine & Rehabilitation.* 91: S270–S279.

70. Shimizu,Y., Kadone, H., Kubota, S., Ikumi, A., Abe, T., Marushima, A., Ueno, T., Endo, A., Kawamoto, H., Saotome, K., Matsushita, A., M., Matsumura, Sankai, Y., Hada, Y. & Yamazaki, M. 2017. Active elbow flexion is possible in C4 quadriplegia using hybrid assistive limb (HAL®) technology: A case study. *Journal of Spinal Cord Medicine.* 40(4):456-462.

71. Trampisch, U.S., Franke, J., Jedamzik, N., Hinrichs, T. & Platen, P. 2012. Optimal Jamar dynamometer handle position to assess maximal isometric hand grip strength in epidemiological studies. *Journal of Hand Surgery*. 37(11), 2368-2373.

72. Wæver, D., Madsen, M. L., Rölfing, J. H. D., Borris, L. C., Henriksen, M., Nagel, L. L. & Thorninger, R. 2018. Distal radius fractures are difficult to classify. *Injury, Int. J. Care Injured*. 49(1): 29-32.

73. Wagner, T. H., Lo, A. C., Peduzzi, P., Bravata, D. M., Huang, G. D., Krebs, H. I., Ringer, R. J., Federman, D. G., Richards, L. G., Haselkorn, J. K., Wittenberg, G. F., Volpe, B. T., Bever, C. T., Duncan, P.W., Siroka, A. & Guarino, P. D. 2011. An economic Analysis of robot-assisted therapy for long-term upper-limb impairment after stroke. *Stroke*. 42:2630-2632.

74. Waljee, J. F., Ladd, A., MacDermid, J., Rozental, T. D., Wolfe, S. W. & Distal Radius Outcomes Consortium. 2016. A unified approach to outcomes assessment for distal radius fractures. *Journal of Hand Surgery*. 41 (4): 565-573.

75. Wan, Y. C., Wickstrom, R., Yen, S. C., Kapellusch, J. & Grogan, K. A. 2018. Assessing manual dexterity: comparing the work ability rate of manipulation test with the Minnesota manual dexterity test. *Journal of Hand Therapy*. 31(3):339-347.

76. Weinstock-Zlotnick, G. & Mehta, S. P. 2016. A structured literature synthesis of wrist outcome measures: An evidence-based approach to determine use among common wrist diagnoses. *Journal of Hand Therapy*. 29: 98-110.

77. Wiemer, P., K€oster, G. & Felderhoff, J., 1992. Fractures of the distal radius. Changing therapeutic strategies. *Orthopade*. 28:846-852.

78. Wu, C. Y., Yang, C. L. & Chuang, L. L., Lin, K. C., Chen, H. C., Chen, M. D. & Huang, W. C. 2012 Effect of therapist-based versus robot-assisted bilateral

arm training on motor control, functional performance, and quality of life after chronic stroke: a clinical trial. *Physical Therapy.* 92:1006–1016.

79. Zhang, C., Li-Tsang, C. W. P. & Au, R. K. C. 2017. Robotic approaches for the rehabilitation of upper limb recovery after stroke: a systematic review and meta-analysis. *International Journal of Rehabilitation Research.* 40:19–28.

Appendix A:

Cyberdyne Hybrid Assistive Limb (HAL) Operator and Instructor Certification

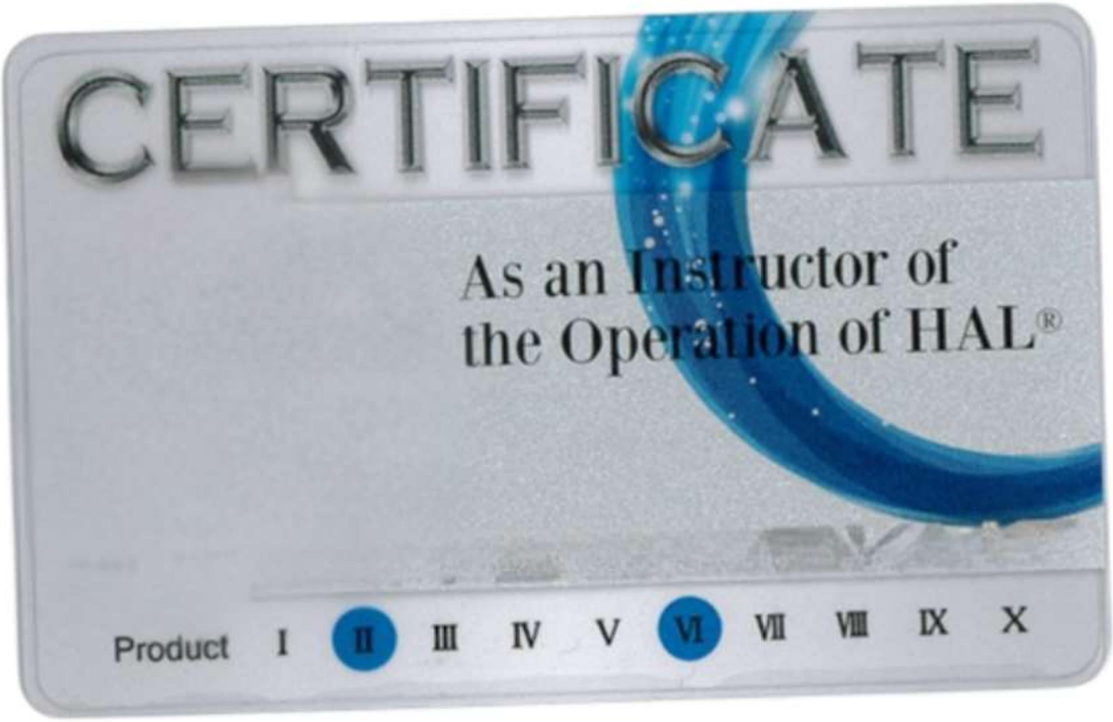

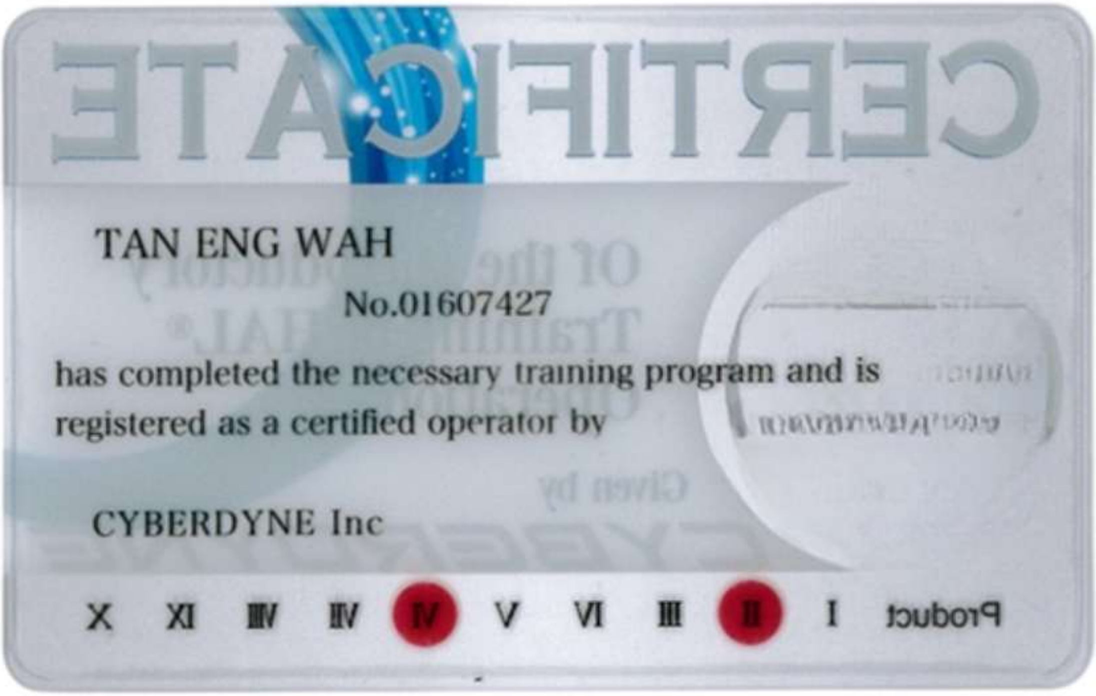

Appendix B: Sociodemographic Questionnaire

THE EFFECTIVENESS OF SINGLE JOINT HYBRID ASSISTIVE LIMB (HAL-SJ) ROBOTIC EXOSKELETON IN IMPROVING FUNCTIONAL OUTCOMES AMONG WORKERS WITH WRIST FRACTURES: A RANDOMIZED CONTROLLED TRIAL

SOCIODEMOGRAPHIC QUESTIONNAIRE

ID: _______________ Date: _____________

Please complete the following items by providing the answers in the space provided:	
Age	
Gender	Male / Female
Race	Malay/ Chinese/ Indian/ Others (please specify)
Marital Status	Married/ Single/ Divorced
Occupation	
Educational Level	UPSR/ PMR/ SPM/ STPM/ Certificate/ Diploma/ Bachelor's Degree/ Master's Degree/ PhD<br>No formal education
Hand Dominance	Right/ Left/ Ambidextrous
Diagnosis	
Date of Injury	
Date of Referral	
History of Present Injury	
Mechanism of injury	
Medical/ Surgical Management	Conservative/ Operative/ Conservative & Operative
Types of Medical/ Surgical Interventions	
Date of Surgery	
Types of Wrist Fracture	Extra-articular/ Partial Articular/ Intra-Articular
Employment Status	Employed/ Unemployed
Monthly Salary	
Medical Leave	Yes / No
SOCSO Compensation Scheme	Employment Injury / Invalidity

Appendix C: Disabilities of the Arm, Shoulder and Hand Outcome Measure

DISABILITIES OF THE ARM, SHOULDER AND HAND

Please rate your ability to do the following activities in the last week by circling the number below the appropriate response.

	NO DIFFICULTY	MILD DIFFICULTY	MODERATE DIFFICULTY	SEVERE DIFFICULTY	UNABLE
1. Open a tight or new jar.	1	2	3	4	5
2. Write.	1	2	3	4	5
3. Turn a key.	1	2	3	4	5
4. Prepare a meal.	1	2	3	4	5
5. Push open a heavy door.	1	2	3	4	5
6. Place an object on a shelf above your head.	1	2	3	4	5
7. Do heavy household chores (e.g., wash walls, wash floors).	1	2	3	4	5
8. Garden or do yard work.	1	2	3	4	5
9. Make a bed.	1	2	3	4	5
10. Carry a shopping bag or briefcase.	1	2	3	4	5
11. Carry a heavy object (over 10 lbs).	1	2	3	4	5
12. Change a lightbulb overhead.	1	2	3	4	5
13. Wash or blow dry your hair.	1	2	3	4	5
14. Wash your back.	1	2	3	4	5
15. Put on a pullover sweater.	1	2	3	4	5
16. Use a knife to cut food.	1	2	3	4	5
17. Recreational activities which require little effort (e.g., cardplaying, knitting, etc.).	1	2	3	4	5
18. Recreational activities in which you take some force or impact through your arm, shoulder or hand (e.g., golf, hammering, tennis, etc.).	1	2	3	4	5
19. Recreational activities in which you move your arm freely (e.g., playing frisbee, badminton, etc.).	1	2	3	4	5
20. Manage transportation needs (getting from one place to another).	1	2	3	4	5
21. Sexual activities.	1	2	3	4	5

Disabilities of the Arm, Shoulder and Hand

	NOT AT ALL	SLIGHTLY	MODERATELY	QUITE A BIT	EXTREMELY
22. During the past week, *to what extent* has your arm, shoulder or hand problem interfered with your normal social activities with family, friends, neighbours or groups? *(circle number)*	1	2	3	4	5

	NOT LIMITED AT ALL	SLIGHTLY LIMITED	MODERATELY LIMITED	VERY LIMITED	UNABLE
23. During the past week, were you limited in your work or other regular daily activities as a result of your arm, shoulder or hand problem? *(circle number)*	1	2	3	4	5

Please rate the severity of the following symptoms in the last week. *(circle number)*

	NONE	MILD	MODERATE	SEVERE	EXTREME
24. Arm, shoulder or hand pain.	1	2	3	4	5
25. Arm, shoulder or hand pain when you performed any specific activity.	1	2	3	4	5
26. Tingling (pins and needles) in your arm, shoulder or hand.	1	2	3	4	5
27. Weakness in your arm, shoulder or hand.	1	2	3	4	5
28. Stiffness in your arm, shoulder or hand.	1	2	3	4	5

	NO DIFFICULTY	MILD DIFFICULTY	MODERATE DIFFICULTY	SEVERE DIFFICULTY	SO MUCH DIFFICULTY THAT I CAN'T SLEEP
29. During the past week, how much difficulty have you had sleeping because of the pain in your arm, shoulder or hand? *(circle number)*	1	2	3	4	5

	STRONGLY DISAGREE	DISAGREE	NEITHER AGREE NOR DISAGREE	AGREE	STRONGLY AGREE
30. I feel less capable, less confident or less useful because of my arm, shoulder or hand problem. *(circle number)*	1	2	3	4	5

DASH DISABILITY/SYMPTOM SCORE = _____ ([(sum of n responses / n) - 1] x 25, where n is the number of completed responses.)

A DASH score may not be calculated if there are greater than 3 missing items.

Appendix D: Disabilities of the Arm, Shoulder and Hand Outcome Measure (Malay Version)

TANGAN, BAHU DAN LENGAN KURANG UPAYA

Sila nyatakan kebolehan anda melakukan aktiviti-aktiviti pada minggu lepas dengan membulatkan nombor berdasarkan nombor berdasarkan jawapan yang sesuai.

	Mudah	Kurang mudah	Sederhana susah	Sangat susah	Tidak boleh langsung
1. Membuka penutup balang yang baru atau yang ketat.	1	2	3	4	5
2. Menulis.	1	2	3	4	5
3. Memutar kunci.	1	2	3	4	5
4. Menyediakan makanan.	1	2	3	4	5
5. Menolak pintu yang berat.	1	2	3	4	5
6. Meletakkan barang di atas rak yang lebih tinggi daripada kepala anda.	1	2	3	4	5
7. Melaksanakan kerja-kerja rumah yang berat (cth. Membasuh dinding lantai).	1	2	3	4	5
8. Berkebun.	1	2	3	4	5
9. Mengemas tempat tidur.	1	2	3	4	5
10. Membimbit bakul membeli-belah atau beg bimbit.	1	2	3	4	5
11. Membawa barang berat (melebihi 10 paun/ 4.5 kg).	1	2	3	4	5
12. Mengganti mentol lampu siling.	1	2	3	4	5
13. Mencuci atau mengeringkan rambut anda.	1	2	3	4	5
14. Membasuh belakang tubuh.	1	2	3	4	5
15. Menyarung baju.	1	2	3	4	5
16. Menggunakan pisau untuk memotong makanan.	1	2	3	4	5
17. Aktiviti-aktiviti rekreasi yang memerlukan daya yang rendah (cth. Bermain kad terup, mengait, dll.)	1	2	3	4	5
18. Melakukan aktiviti rekreasi di mana anda terpaksa menggunakan daya atau impak tangan, bahu atau lengan (cth. Bermain golf, bermain, tenis, menukul dll.)	1	2	3	4	5
19. Aktiviti-aktiviti rekreasi yang memerlukan anda menggerakkan lengan secara bebas (bermain piring terbang '*frisbee*', badminton, dll.)	1	2	3	4	5
20. Kemampuan untuk bergerak (bergerak dari satu tempat ke satu tempat).	1	2	3	4	5
21. Aktiviti seksual.	1	2	3	4	5

TANGAN, BAHU DAN LENGAN KURANG UPAYA

	Tidak pernah	Jarang-jarang	Kadang-kadang	Selalu	Sentiasa
22. Pada minggu lepas, setakat mana masalah tangan, bahu atau lengan anda mengganggu aktiviti sosial harian anda bersama keluarga, kawan-kawan, jiran atau kumpulan? (bulatkan nombor)	1	2	3	4	5

	Tiada halangan	Sedikit terhalang	Sederhana terhalang	Sangat terhalang	Tidak boleh langsung
23. Pada minggu lepas, adakah pekerjaan atau aktiviti-aktiviti harian yang lain terhalang disebabkan oleh masalah tangan, bahu atau lengan?(bulatkan nombor)	1	2	3	4	5

Sila nyatakan tahap keterukan simptom-simptom berikut pada minggu lepas. (Bulatkan nombor)

	Tiada	Sedikit	Sederhana	Teruk	Sangat teruk
24. Sakit tangan, bahu atau lengan. (semasa berehat)	1	2	3	4	5
25. Sakit tangan, bahu atau lengan apabila melakukan aktiviti-aktiviti tertentu.	1	2	3	4	5
26. Sesemut (menyucuk-nyucuk) pada tangan, bahu atau lengan.	1	2	3	4	5
27. Rasa lemah pada tangan, bahu atau lengan.	1	2	3	4	5
28. Rasa kaku pada tangan, bahu atau lengan.	1	2	3	4	5

	Mudah	Kurang mudah	Sederhana susah	Sangat susah	Terlalu sukar sehingga saya tidak dapat tidur
29. Pada minggu lepas, nyatakan kesukaran anda untuk tidur akibat kesakitan pada tangan, bahu atau lengan?(bulatkan nombor)	1	2	3	4	5

	Sangat tidak setuju	Tidak setuju	Tidak pasti	Setuju	Sangat setuju
30. Saya berasa tidak ada kemampuan, tidak yakin, atau tidak berguna disebabkan oleh masalah tangan, bahu atau lengan.	1	2	3	4	5

KURANG UPAYA DASH/ SKOR SIMPTOM = $\left(\left[\frac{\text{jumlah dari nilai yang dipilih}}{\text{soalan dijawab}}\right]-1\right)\times 25$

Skor DASH <u>tidak boleh</u> dikira jika terdapat lebih daripada 3 butiran yang tidak dijawab.

Appendix E: Disabilities of the Arm, Shoulder and Hand Outcome Measure (Simplified Chinese Version)

上肢功能障碍评定量表

请根据过去一个星期您从事如下活动的能力来打分，在下面合适的分数上画圈。

		毫无困难	有点困难	中度困难	非常困难	无法完成
1.	打开一个紧的或新的罐子。	1	2	3	4	5
2.	书写。	1	2	3	4	5
3.	转动钥匙（开锁、发动引擎）。	1	2	3	4	5
4.	准备一顿饭。	1	2	3	4	5
5.	推开一扇沉重的门。	1	2	3	4	5
6.	在高过您头顶的架子上放置物品。	1	2	3	4	5
7.	做繁重的家务活（如： 刷墙，擦地板）。	1	2	3	4	5
8.	种植或整理园子。	1	2	3	4	5
9.	铺床。	1	2	3	4	5
10.	提购物袋或公文包。	1	2	3	4	5
11.	提重物（超过4.5千克）。	1	2	3	4	5
12.	换高过头顶的灯泡。	1	2	3	4	5
13.	洗或吹干您的头发。	1	2	3	4	5
14.	洗后背。	1	2	3	4	5
15.	穿套头毛衣。	1	2	3	4	5
16.	用刀子切食物。	1	2	3	4	5
17.	几乎不需要费力就能完成的休闲活动（如：打扑克牌，织毛线等）。	1	2	3	4	5
18.	需要手，手臂或肩膀使用些力量才能完成的休闲活动（如：高尔夫球、使用锤子做活、网球等）。	1	2	3	4	5
19.	需要灵活使用手臂才能完成的休闲活动（如：玩飞盘，打羽毛球等）。	1	2	3	4	5
20.	完成交通需求（从一个地方到另一个地方）。	1	2	3	4	5
21.	性活动。	1	2	3	4	5

上肢功能障碍评定量表

	一点儿也不	轻度	中度	重度	极度
22. 在过去的一星期内，您的手，手臂或肩部对您在与家人，朋友，邻居和组群的正常社交活动中产生了何种程度的影响？（在数字上画圈）	1	2	3	4	5

	毫不受限	轻度受限	中度受限	非常受限	不能完成
23. 在过去的一星期中，您的手，手臂或肩部问题是否限制了您的工作或者日常活动？（在数字上画圈）	1	2	3	4	5

请为过去一个星期里下列症状的严重程度打分。（在数字上画圈）

	无	轻度	中度	重度	极度
24. 手臂，肩部或手疼痛。	1	2	3	4	5
25. 当进行某项特定活动时手臂，肩部或手疼痛。	1	2	3	4	5
26. 手臂，肩部或手的刺痛感（针刺样）。	1	2	3	4	5
27. 手臂，肩部，手无力。	1	2	3	4	5
28. 手臂，肩部，手僵硬。	1	2	3	4	5

	毫无困难	有点困难	中度困难	非常困难	太困难了以致于我无法入睡
29. 在过去的一星期中，由于您手臂，肩部或手疼痛给您带来了何种程度的睡眠困难？（在数字上画圈）	1	2	3	4	5

	强烈不赞同	不赞同	不赞同也不反对	赞同	强烈赞同
30. 由于手臂，肩部，手的问题，我觉得能力很差，很没自信，很没用。（在数字上画圈）	1	2	3	4	5

DASH功能障碍/症状得分=[（n个作答得分的平均分）-1]×25，n代表已答题目的数量
如果有3个以上遗漏项目，DASH分数不予计算。

Chinese (Simplified) translation courtesy of Dr. Le Qi, Hand Surgery, China-Japan Union Hospital of Jilin University, China.

Appendix F: Lam Assessment of Stages of Employment Readiness (LASER)

Name: ……………………………………… Gender: ☐ Male ☐ Female
Date: ……………………….

	This questionnaire is to help us better understanding your needs. Each statement describes how a person might feel when staring a job service program with good will Industries. Please indicate the level of agreement you have with each statement. In case, make your choice in term of how you feel right now, not what you have felt in the past or would like to feel.	Strongly Disagree<br>1	Disagree<br>2	Undecided<br>3	Agree<br>4	Strongly Agree<br>5
1	I think I might be ready to look for some kind of job.					
2	I am doing something to get ready to look for a job					
3	It might be worthwhile to work on finding a job					
4	I am not able to work. I do not see why I have to be here.					
5	I am finally doing something about finding a job.					
6	I have been thinking that it might be time for me to find a job.					
7	Getting myself ready to find a job is pretty much a waste of time because I can ‘t work anyway.					
8	I guess being out of work is not good, but there is nothing I can do about it right now.					
9	I know I need to get a job and really think I should work on finding one.					
10	People tell me that I should get a job, but I don’t think so.					
11	Anyone can talk about wanting to find a job, but I am actually doing something about it.					
12	All this talk about work is boring. Why can’t people just leave me alone?					
13	I am actively doing something to find a job					
14	It is a pretty much a waste of time getting ready to find a job because I ready don’t want to work.					

Lam Assessment of Stages of Employment Readiness (LASER)
Scoring Sheet

Date of Assessment:

PRE CONTEMPLATION STAGE	COMTEMPLATION	ACTION STAGE
4	1	2
7	3	5
8	6	11
10	9	13
12		
14		
Total	Total	Total

Date of Assessment:

PRE CONTEMPLATION STAGE	COMTEMPLATION	ACTION STAGE
4	1	2
7	3	5
8	6	11
10	9	13
12		
14		
Total	Total	Total

Appendix G: Lam Assessment of Stages of Employment Readiness (LASER) (Malay Version)

PENILAIAN PERINGKAT KESEDIAAN PEKERJAAN LAM (L.A.S.E.R)

Nama: ____ Jantina: __________________

Tarikh: ___________________________

Borang soal selidik ini adalah untuk membantu lebih memahami keperluan klien. Setiap pernyataan menerangkan bagaimana perasaan seseorang itu mungkin apabila memulakan program perkhidmatan pekerjaan dengan industri yang muhibah. Sila nyatakan tahap persetujuan anda dengan setiap kenyataan berikut. Dalam setiap kes, sila buat pilihan anda berdasarkan terma seperti bagaimana perasaan anda sekarang, bukan apa yang anda telah dirasai di masa lalu atau ingin rasai.

	**Sangat tidak setuju**	**Tidak setuju**	**Tidak pasti**	**setuju**	**Sangat bersetuju**
1. Saya rasa mungkin saya sudah bersedia untuk mencari beberapa jenis pekerjaan					
2. Saya melakukan sesuatu untuk mendapatkan pekerjaan					
3. Ia mungkin agak berbaloi untuk mencari pekerjaan					
4. Saya tidak mampu bekerja. Saya tidak nampak mengapa saya perlu mencari pekerjaan					
5. Saya akhirnya melakukan sesuatu untuk mendapatkan pekerjaan					
6. Saya telah berfikir bahawa ia mungkin adalah masa yang sesuai bagi saya untuk mencari pekerjaan					
7. Mempersiapkan diri saya untuk mencari pekerjaan adalah sangat membuang masa kerana saya sudah tidak boleh bekerja					
8. Saya rasa tidak mempunyai pekerjaan dalam tempoh yang agak lama adalah tidak bagus, tetapi tiada apa yang boleh saya lakukan sekarang					

9. Saya tahu saya perlu untuk mendapatkan pekerjaan dan saya fikir patut untuk mencari satu pekerjaan					
10. Orang memberitahu saya bahawa saya perlu mendapatkan pekerjaan, tetapi saya tidak fikir begitu					
11. Sesiapa sahaja boleh bercakap tentang mahu untuk mencari pekerjaan, tetapi saya benar-benar melakukan sesuatu mengenainya					
12. Semua perbincangan ini adalah membosankan. Mengapa orang lain tidak meninggalkan saya sendirian sahaja?					
13. Saya secara aktif melakukan sesuatu untuk mencari pekerjaan					
14. Ia adalah sesuatu yang membuang masa bersedia untuk mencari pekerjaan kerana saya benar-benar tidak mahu bekerja lagi					

PENILAIAN PERINGKAT KESEDIAAN PEKERJAAN LAM (L.A.S.E.R)
(borang pemarkahan)

Tarikh Penilaian:

**Peringkat Pra-Jangkaan**	**Peringkat Jangkaan**	**Peringkat Tindakan**
**4.**	**1.**	**2.**
**7.**	**3.**	**5.**
**8.**	**6.**	**11.**
**10.**	**9.**	**13.**
**12.**		
**14.**		
**Jumlah:**	**Jumlah:**	**Jumlah**

Tarikh Penilaian:

**Peringkat Pra-Jangkaan**	**Peringkat Jangkaan**	**Peringkat Tindakan**
**4.**	**1.**	**2.**
**7.**	**3.**	**5.**
**8.**	**6.**	**11.**
**10.**	**9.**	**13.**
**12.**		
**14.**		
**Jumlah:**	**Jumlah:**	**Jumlah**

Appendix H: Lam Assessment of Stages of Employment Readiness (LASER) (Chinese Version)

姓名：______________ 性別：❑男 ❑女 今天日期：____________ 出生日期：____________

**此問卷可幫助我們更了解你的需要。每句句子描述了一個人開始求職服務計劃時的感覺。請在適當的方格用剔號（✓）指出你對每句句子的同意程度。請依照你現在的感覺去決定您/你的選擇，而非您/你過去或將來的感覺。**		☹ **非常不同意**	🙁 **不同意**	😐 **不確定**	🙂 **同意**	☺ **非常同意**
1.	我覺得我已經準備好去搵一份工作。					
2.	我正開始行動去準備搵工。					
3.	我認爲花功夫在搵工上是值得的。					
4.	我根本無工作能力，我不明白爲何要參加這計劃。					
5.	我終於開始爲搵工做一些功夫。					
6.	我想現在是我搵工的適當時機。					
7.	我根本沒有工作能力，所以爲自己做好準備去搵工是浪費時間的。					
8.	雖然我覺得無工做是不太好，但現在我是無能爲力。					
9.	我知我需要搵一份工，我亦認爲我必須努力搵工。					
10.	其他人告訴我應該要搵工，但我並不同意。					
11.	任何人都可以口講自己想搵工，但我就不同，我實際上正在努力搵工。					
12.	所有這些關於搵工的問題都好悶，爲什麼不讓我自己一個人靜一靜？					
13.	我正在積極地搵工。					
14.	我根本不想返工，爲搵工做準備工夫根本是浪費時間。					

Appendix I: Visual Analogue Scale (VAS)

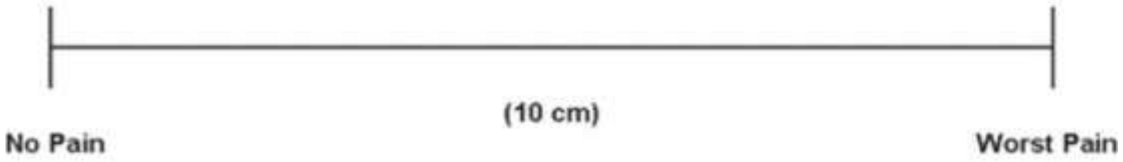

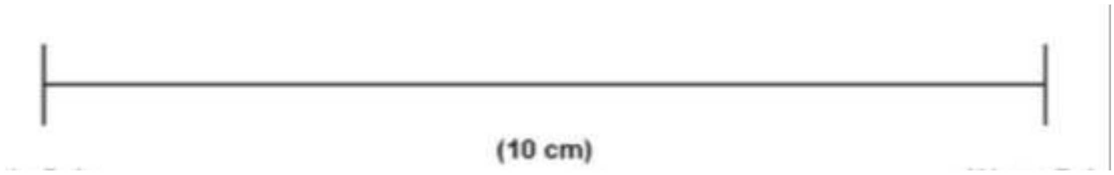

Tiada Sakit **Sakit Yang Paling Teruk**

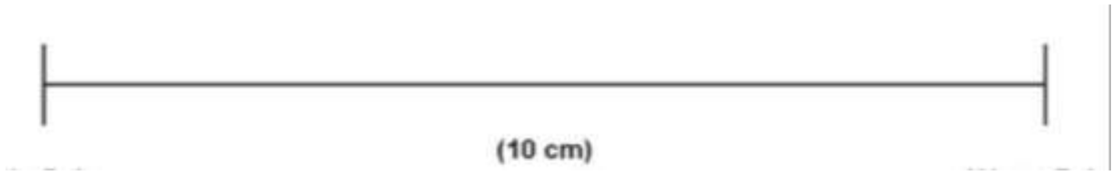

没有痛楚 非常痛楚

Appendix J: CLINICAL DATA RECORD SHEET

1) Disabilities of the Arm, Shoulder and Hand (DASH) Outcome Measure

	Initial Assessment<br>Date:	Reassessment<br>Date:
DASH Symptom Score		

2) LAM Assessment on Stages of Employment Readiness (LASER) Questionnaire

	Initial Assessment<br>Date:	Reassessment<br>Date:
LASER Score		

3) Visual Analogue Scale (VAS)

	Initial Assessment<br>Date:	Reassessment<br>Date:
VAS Score		

4) Grip Strength

	Initial Assessment<br>Date:	Reassessment<br>Date:
1st Trial		
2nd Trial		
3rd Trial		
Average		

5) Lateral Pinch Strength

	Initial Assessment<br>Date:	Reassessment<br>Date:
1st Trial		
2nd Trial		
3rd Trial		
Average		

6) Tripod Pinch Strength

	Initial Assessment<br>Date:	Reassessment<br>Date:
1st Trial		
2nd Trial		
3rd Trial		
Average		

7) Tip-to-tip Pinch Strength

	Initial Assessment<br>Date:	Reassessment<br>Date:
1st Trial		
2nd Trial		
3rd Trial		
Average		

8) Active Range of Motion – Forearm

	Initial Assessment<br>Date:	Reassessment<br>Date:
Supination		
Pronation		

9) Active Range of Motion – Wrist

	Initial Assessment<br>Date:	Reassessment<br>Date:
Extension		
Flexion		
Radial Deviation		
Ulnar Deviation		

10) Active Range of Motion – Hand

		Initial Assessment<br>Date:	Reassessment<br>Date:
Thumb	MCPJ Flexion		
	IPJ Flexion		
Index Finger	MCPJ Flexion		
	PIPJ Flexion		
	DIPJ Flexion		
Middle Finger	MCPJ Flexion		
	PIPJ Flexion		
	DIPJ Flexion		
Ring Finger	MCPJ Flexion		
	PIPJ Flexion		
	DIPJ Flexion		
Little Finger	MCPJ Flexion		
	PIPJ Flexion		
	DIPJ Flexion		

11) Purdue Pegboard Test

Initial Assessment Date:

	1st Trial	2nd Trial	3rd Trial	Average
Right Hand				
Left Hand				
Both Hand				
R + L + B				
Assembly				

Reassessment Date:

	1st Trial	2nd Trial	3rd Trial	Average
Right Hand				
Left Hand				
Both Hand				
R + L + B				
Assembly				

12) Box and Block Test

Initial Assessment Date:

	Right Hand	Left Hand
Number of Blocks Transferred in 60 Seconds		

Reassessment Date:

	Right Hand	Left Hand
Number of Blocks Transferred in 60 Seconds		

Appendix K

INFORMATION SHEET

RANDOMIZED CONTROLLED TRIAL

Research title:
The Effectiveness of Single Joint Hybrid Assistive Limb (HAL-SJ) Robotic Exoskeleton in Improving Functional Outcomes among Workers with Wrist Fractures: A Randomized Controlled Trial

Introduction

You are invited to participate in this research study because you have sustained a wrist fracture that had limited your performance in daily and work activities. Wrist fracture cases can take up to few months or even one to two years to achieve maximal functional improvement. The residual disability or pain, along with the long functional recovery period following wrist fracture may impact the socioeconomics of a person, family, and society significantly. Thus, a new intervention with the robotic exoskeleton will be implemented along with the standard hand therapy program to optimize the functional recovery process.

The details of the study will be described in this document. It is important for you to fully understand the purpose of this study and what it will involve. Kindly read through this information thoroughly for your consideration and decision to participate in this study. Ask the researcher if there is anything that is unclear or if you would like more information or clarification. Take time to decide whether or not you wish to take part.

Participation in this study is voluntary. If you agree to take part, then you will be asked to sign the "Informed Consent Form". You will be given a copy of the consent form and this Information Sheet. If you do not agree to participate, you do not need to give any reason and nothing will be affected. If you decided to participate, you can still withdraw from the study at any time without any penalty. If you withdraw, any data collected from you up to your withdrawal will still be used for the study. Your refusal to participate or withdrawal will not affect any medical or health benefits to which you are

otherwise entitled. The researcher may also remove you from the study for a variety of reason.

Purpose of study

The purpose of this study is to evaluate the effectiveness of single joint hybrid assistive limb (HAL-SJ) robotic exoskeleton in improving the functional outcomes among workers with wrist fractures. The application of robotic technology in facilitating upper limb movement and functional recovery training is extensive in the field of neurorehabilitation but limited in the field of orthopaedic rehabilitation particularly in hand therapy practice.

Robotic interventions had been proved to provide motor control/learning, practice-induced neuroplasticity, intensity, and task specific training for greater functional recovery. The upper limb HAL-SJ is a wearable movement support robot that detects bioelectrical signals on the skin surface and assists joint movements by controlling and operating an actuator placed outside the respective joint based on the frontier science “Cybernics”. Currently, there are vast of studies on the clinical applications of robotic interventions for neurological rehabilitation. However, the use of robotic interventions in orthopaedic rehabilitation especially for the wrist and hand remains inadequately explored. Therefore, this study aims to determine the effectiveness of robotic interventions in improving the functions as well as the recovery period following wrist fracture.

A total of 38 participants who sustained wrist fractures as similar to you will be recruited to participate in this study. Your participation in this study will only last for 4 weeks while the whole study will be implemented for 36 months.

What will the study involve?

If you agree to participate in the study, you will be asked to complete a series of questionnaires and complete a few measurements. You will then be randomly assigned to one of the treatment groups below by drawing from concealed envelopes. You have equal chance of being assigned to each of the groups.

Group 1:

a) Participants will undergo two conventional therapy sessions daily that include an occupational therapy session and a physiotherapy session on a 5-day/week routine basis.

Group 2:

a) Participants will undergo two conventional therapy sessions daily that include an occupational therapy session and a physiotherapy session on a 5-day/week routine basis.
b) Within two hours after the conventional therapy sessions, participants will undergo the HAL-SJ robotic therapy session conducted by the researcher at a separate treatment room on a 5-day/week routine basis.

Regardless of your group allocation, you will need to complete the following questionnaires and measurements administered by the therapist at the beginning of the hand therapy treatment (pre-intervention/initial assessment) and after 4 weeks of hand therapy treatment (post-intervention):

1) Functional performance by using Disabilities of the Arm, Shoulder and Hand (DASH) Outcome Measure
2) Work readiness by using LAM Assessment on Stages of Employment Readiness (LASER) Questionnaire
3) Pain level by using Visual Analogue Scale (VAS)
4) Physical performance of Active Range of Motion (AROM) of the hand and wrist by using goniometer
5) Physical performance of grip and pinch strength by using Jamar Dynamometer and B&L Engineering Pinch Gauge respectively
6) Physical performance of fine dexterity by using Purdue Pegboard Test (PPT)
7) Physical performance of gross manual dexterity by using Box and Block Test (BBT)

Post-intervention outcome measurements will be performed after the 20th therapy session, i.e., upon the completion of the 4-weeks program.

It is important that you answer all of the questions asked by the therapist honestly and completely. You must inform the therapist if your condition or circumstances change during the study or if you make any changes to any of your current treatments.

After the trial, you will continue receiving the necessary therapy sessions as usual. Whether you complete the study or withdraw early, the best alternatives for your future treatment will be discussed with the treating team.

Risks and benefits:

Single Joint Hybrid Assistive Limb (HAL-SJ) robotic exoskeleton is certified by ISO 13485:2016 (certificate number: 1757.181211) and EC (registration number: DD 601417310001) for rehabilitation and physical therapy usage.

The potential risks of participating in the Single Joint Hybrid Assistive Limb (HAL-SJ) robotic exoskeleton session may include:

a) Skin allergy or reddening of area where electrode is affixed. However, the reddening will disappear shortly after the electrode was taken off
b) Abrasion of areas that contact the device e.g. cuffs and straps
c) Muscle and joint soreness (due to post exercise effect)

The researcher of this study, who is a certified HAL-SJ operator accredited by Cyberdyne Inc. will take necessary precaution and careful supervision throughout the entire Single Joint Hybrid Assistive Limb (HAL-SJ) robotic exoskeleton session to minimise these potential risks. You are advised to communicate and inform the researcher immediately if you have any concern of the above risks.

By participating in this study, you will understand the performance of your physical and psychological functions before and after the therapy program. If you are being allocated to the intervention group that receives an extra Single Joint Hybrid Assistive Limb (HAL-SJ) robotic exoskeleton session, your performance of physical and psychological functions will facilitate our understanding regarding the therapeutic effects of Single Joint Hybrid Assistive Limb (HAL-SJ) robotic exoskeleton in wrist fracture rehabilitation.

Do you have to take part?

Participation in this study is voluntary. If you agree to take part, then you will be asked to sign the "Informed Consent Form". You will be given a copy of the consent form and this Information Sheet. Throughout your participation, you may also refuse to answer any questions that you do not want to answer. If you are not agreeing to participate, you do not need to give any reason and nothing will be affected. If you decided to participate, you can still withdraw from the study at any time without any penalty. If you withdraw, any data collected from you up to your withdrawal will still be used for the study. Your refusal to participate or withdrawal will not affect any medical or health benefits to which you are otherwise entitled.

Data and confidentiality

All your information obtained in this study will be kept and handled in a confidential manner, in accordance with applicable laws and/or regulations. The data from this study will be made into a report which may be published. Once the data have been gathered and the research completed, the raw materials will be kept in the locked file cabinet in the office of the researcher. Electronic data will be securely kept using password. Access to the data is only by the researcher. After 5 years, the material will be shredded and discarded. The data will be reported in a collective manner with no reference to an individual.

Payment and compensation

This study is self-funded by the researcher himself. No external funding or grant is received. Therefore, no payment will be given to you for participating in this study. If you are allocated into Group 2 (intervention group), you will not be charged for the additional Single Joint Hybrid Assistive Limb (HAL-SJ) robotic exoskeleton sessions received.

Can this study or my participation be terminated early?

The researcher may stop the study or your participation at any time due to the concerns of your safety. If the study is stopped early for any reason, you will be informed and arrangements will be made for your future care. You will have the opportunity to see your own measurement results upon completion of this study.

Who should I call if I have questions?

If you have any questions about the study or if you think you have a study related injury and you want information about treatment, please contact the following researchers:

Dr. Chai Siaw Chui
Main Supervisor
Occupational Therapy Programme
Faculty of Health Sciences
Universiti Kebangsaan Malaysia
Telephone No.: 03-92897047
Email: sc.chai@ukm.edu.my

Mr. Tan Eng Wah
Master’s Candidate
Occupational Therapy Programme
Faculty of Health Sciences
Universiti Kebangsaan Malaysia
Telephone No.: 012-6358531
Email: tanew9911@gmail.my

INFORMED CONSENT FORM

Title of Study: The Effectiveness of Single Joint Hybrid Assistive Limb (HAL-SJ) Robotic Exoskeleton in Improving Functional Outcomes among Workers with Wrist Fractures: A Randomized Controlled Trial

By signing below I confirm the following:

- I have been given oral and written information for the above study and have read and understood the information given.
- I have had sufficient time to consider participation in the study and have had the opportunity to ask questions and all my questions have been answered satisfactorily.
- I understand that my participation is voluntary and I can withdraw from the study at any time without giving a reason and this will not affect my future treatment. I am not taking part in any other research study at this time. I understand the risks and benefits, and I freely give my informed consent to participate under the conditions stated. I understand that I must follow the researcher instructions related to my participation in the study.
- All personal details will be treated as CONFIDENTIAL.
- I will receive a copy of this subject information/informed consent form signed and dated to bring home.

Subject:

Signature:	I/C number:
Name:	Date:

<u>Investigator conducting informed consent:</u>

Signature: I/C number:

Name: Date:

<u>Impartial witness:</u>

Signature: I/C number:

Name: Date:

<u>HELAIAN MAKLUMAT</u>

PERCUBAAN KAWALAN RAWAK

Tajuk Kajian:

Keberkesanan Eksoskeletal Robotik *Single Joint Hybrid Assistive Limb (HAL-SJ)* dalam Meningkatkan Kefungsian di Kalangan Pekerja yang Mengalami Kepatahan Tulang Pergelangan Tangan: Percubaan Kawalan Rawak

Pengenalan

Anda telah dijemput untuk menyertai kajian penyelidikan ini kerana anda telah mengalami kepatahan tulang pergelangan tangan yang mengehadkan tahap prestasi anda dalam aktitivi-aktiviti harian dan kerja. Kes-kes kepatahan tulang pergelangan tangan mengambil tempoh masa beberapa bulan atau satu hingga dua tahun untuk mencapai perkembangan kefungsian yang maksima. Kekurangan keupayaan dan kesakitan di samping tempoh masa penyembuhan yang panjang akibat daripada kepatahan tulang pergelangan tangan boleh memberikan impak yang ketara terhadap tahap sosioekonomi individu, keluarga dan masyarakat. Maka, suatu intervensi baru dengan eksoskeletal robotik akan digunakan dalam program terapi tangan untuk mengoptimumkan proses pemulihan tahap kefungsian.

Maklumat tentang kajian ini akan dinyatakan dalam dokumen kini. Adalah penting untuk anda memahami secara sepenuhnya tentang tujuan dan perkara yang terlibat dalam kajian ini. Sila baca secara menyeluruh dan mempertimbangkan dengan teliti penerangan yang diberi sebelum anda bersetuju untuk menyertai penyelidikan ini. Jika terdapat sebarang kemusykilan ataupun maklumat lanjut yang anda ingin tahu, anda boleh bertanya kepada terapis yang terlibat dalam penyelidikan ini. Sila ambil masa yang secukupnya untuk membuat keputusan sama ada anda ingin menyertai kajian penyelidikan ini.

Penyertaan dalam penyelidikan ini adalah secara sukarela. Sekiranya anda bersetuju untuk menyertai penyelidikan ini, anda akan dikehendaki untuk menandatangani Borang Persetujuan atau Keizinan Peserta pada muka surat akhir risalah ini. Sekiranya anda tidak bersetuju untuk menyertai penyelidikan ini, anda tidak perlu memberikan

sebarang sebab dan perkara ini tidak akan memberikan sebarang kesan pada anda. Jikalau anda membuat keputusan untuk menyertai penyelidikan ini, anda berhak untuk menarik diri pada bila-bila masa tanpa dikenakan sebarang hukuman. Penolakan atau penarikan diri daripada penyelidikan ini tidak akan menjejaskan sebarang faedah perubatan atau kesihatan yang tertakluk kepada hak anda. Penyelidik kajian juga berhak untuk mengecualikan anda daripada penyelidikan ini atas pelbagai sebab.

Tujuan Kajian

Tujuan penyelidikan ini adalah untuk menilai keberkesanan eksoskeletal robotik of *Single Joint Hybrid Assistive Limb (HAL-SJ)* dalam meningkatan kefungsian dalam kalangan pekerja yang mengalami kepatahan pergalangan tangan. Terdapat banyak aplikasi teknologi robotik digunakan untuk membantu pergerakan anggota atas dan latihan pemulihan kefungsian adalah dalam bidang rehabilitasi penyakit saraf atau sistem saraf. Namun, aplikasi tersebut adalah amat terhad dalam bidang rehabilitasi ortopedik terutamanya terapi tangan. Intervensi robotik adalah terbukti dapat meningkatkan pemulihan kefungsian melalui pengawalan/pembelajaran motor, keplastikan neuro atau saraf, intensiti dan latihan aktiviti khusus. HAL-SJ anggota atas merupakan sejenis robot yang dapat dipakai untuk menyokong pergerakan. HAL-SJ anggota atas dapat mengesan isyarat bioelektrik pada permukaan kulit dan membantu pergerakan sendi melalui kawalan dan operasi unit penggerak berdasarkan bidang sains yang dikenali sebagai "*Cybernics*". Kini, terdapat banyak kajian terhadap aplikasi klinikal intervensi robotik bagi populasi yang mengalami masalah atau penyakit sistem saraf. Namun, kajian berkaitan penggunaan intervensi robotik dalam bidang rehabilitasi ortopedik terutamanya bagi bahagian tangan dan pergelangan tangan adalah masih berkurangan. Justeru itu, penyelidikan ini bertujuan untuk menentukan keberkesanan intervensi robotik dalam meningkatkan tahap kefungsian dan tempoh pemulihan berikutan kepatahan pergelangan tangan.

Sebanyak 38 orang peserta yang mengalami kepatahan tulang pergelangan tangan seperti anda akan dijemput untuk mengambil bahagian dalam penyelidikan ini. Penyertaan anda dalam penyelidikan ini hanya akan berlangsung selama 4 minggu sementara keseluruhan kajian penyelidikan ini akan dijalankan selama 36 bulan.

Apakah yang akan terlibat dalam kajian ini?

Sekiranya anda bersetuju untuk menyertai penyelidikan ini, anda akan diminta untuk melengkapkan suatu siri borang kaji selidik dan menyempurnakan beberapa penilaian klinikal. Selepas itu, anda akan diperuntukkan ke dalam salah satu daripada dua kumpulan rawatan secara rawak dengan kaedah cabutan daripada sampul yang tertutup. Anda mempunyai peluang yang sama rata untuk diperuntukkan ke dalam salah satu kumpulan tersebut.

Kumpulan 1:

a) Peserta akan menjalani dua sesi terapi lazim setiap hari, iaitu satu sesi terapi carakerja dan satu sesi fisioterapi mengikut jadual lima hari dalam semingu.

Kumpulan 2:

a) Peserta akan menjalani dua sesi terapi lazim setiap hari, iaitu satu sesi terapi carakerja dan satu sesi fisioterapi mengikut jadual lima hari dalam semingu.

b) Dalam masa dua jam selepas sesi terapi lazim, peserta akan menerima sesi terapi robotik HAL-SJ yang akan dikendalikan oleh penyelidik di bilik rawatan yang berasingan mengikut jadual lima hari seminggu.

Tanpa mengira kumpulan anda, anda akan dikehendaki untuk menyempurnakan borang kaji selidik dan penilaian klinikal berikut yang akan dikendalikan oleh terapis anda pada sesi permulaan untuk rawatan terapi tangan (pra-intervensi/ penilaian awal) dan selepas 4 minggu rawatan terapi tangan (pasca-intervensi):

1) Prestasi tahap kefungsian dengan menggunakan borang kaji selidik *Disabilities of the Arm, Shoulder and Hand (DASH)*
2) Tahap kesediaan bekerja dengan menggunakan borang kaji selidik *LAM Assessment on Stages of Employment Readiness (LASER)*
3) Tahap kesakitan dengan menggunakan *Visual Analogue Scale (VAS)*
4) Prestasi fizikal julat pergerakan aktif sendi tangan dan pergelangan tangan dengan menggunakan *goniometer*
5) Prestasi fizikal kekuatan genggaman dan picitan tangan dengan menggunakan *Jamar Dynamometer* dan *B&L Engineering Pinch Gauge*

6) Prestasi fizikal ketangkasan halus tangan dengan menggunakan *Purdue Pegboard Test (PPT)*
7) Prestasi fizikal ketangkasan kasar tangan dengan menggunakan *Box and Block Test* (BBT)

Penilaian hasil pasca-intervensi akan dilakukan selepas sesi terapi yang ke-20, iaitu selepas penyempurnaan program selama 4 minggu.

Adalah penting bahawa anda menjawab semua soalan yang ditanya oleh terapis secara jujur dan menyeluruh. Anda dikehendaki untuk memaklum kepada terapis anda sekiranya kondisi atau keadaan anda berubah dalam tempoh masa penyelidikan atau anda membuat sebarang perubahan kepada rawatan yang sedia ada.

Selepas tempoh penyelidikan, anda akan meneruskan sesi terapi yang diperlukan seperti biasa. Perancangan rawatan akan dibincangkan bersama pasukan rawatan anda selepas anda menyempurnakan penyelidikan berkenaan mahupun menarik diri daripadanya.

Risiko dan Faedah

Eksoskeletal robotik *Single Joint Hybrid Assistive Limb (HAL-SJ)* telah disahkan oleh ISO 13485:2016 (nombor persijilan: 1757.181211) dan EC (nombor pendaftaran: DD 601417310001) untuk digunakan bagi tujuan rehabilitasi dan terapi fizikal.

Risiko yang berpotensi berlaku semasa penyertaan sesi terapi eksoskeletal *robotik Single Joint Hybrid Assistive Limb (HAL-SJ)* berkemungkinan melibatkan:

a) Alahan kulit atau kemerahan pada bahagian yang dilekatkan dengan elektrod. Walaubagaimanapun, kemerahan berkenaan akan menghilang dalam masa yang singkat selepas elektrod ditanggalkan.
b) Lelasan pada bahagian yang mempunyai kontak dengan peralatan berkenaan iaitu *cuff* dan tali pemasangan
c) Rasa sengal pada otot atau sendi (kesan akibat senaman)

Penyelidik dalam kajian penyelidikan ini, yang juga merupakan seorang pengguna HAL-SJ yang bertauliah dengan persijilan daripada Cyberdyne Inc. akan mengambil

langkah-langkah pencegahan dan pengawasan yang berpatutan sepanjang sesi terapi eksoskeletal robotik *Single Joint Hybrid Assistive Limb (HAL-SJ)* untuk mengurangkan sebarang risiko yang berpotensi berlaku. Anda adalah dinasihati untuk berkomunikasi dan memberitahu penyelidik dengan serta merta sekiranya anda mempunyai sebarang kebimbangan tentang risiko berkenaan.

Dengan menyertai penyelidikan ini, anda akan memahami prestasi kefungsian fizikal dan psikologi anda sebelum dan selepas program terapi. Sekiranya anda diperuntukkan kepada kumpulan intervensi yang menerima sesi terapi eksoskeletal robotik *Single Joint Hybrid Assistive Limb (HAL-SJ)* tambahan, prestasi kefungsian fizikal dan psikologi anda akan memudahkan pemahaman kami mengenai kesan terapeutik eksoskeletal robotik *Single Joint Hybrid Assistive Limb (HAL-SJ)* atas pemulihan kepatahan tulang pergelangan tangan.

Adakah anda perlu menyertai?

Penyertaan dalam kajian ini adalah secara sukarela. Sekiranya anda telah bersetuju untuk mengambil bahagian dalam kajian ini, anda akan dikehendaki untuk menandatangani Borang Persetujuan atau Keizinan Peserta. Anda akan diberi satu salinan Borang Persetujuan atau Keizinan Peserta dan Helaian Maklumat ini. Sepanjang tempoh penyertaan penyelidikan, anda boleh menolak sebarang persoalan yang anda tidak ingin untuk menjawab. Setelah anda bersetuju untuk menyertai penyelidikan ini, anda masih boleh menarik diri pada bila-bila masa sahaja. Sekiranya anda menarik diri, sebarang data yang dikumpul daripada anda masih akan digunakan dalam penyelidikan ini. Penolakan atau penarikan diri daripada penyertaan penyelidikan ini tidak akan menjejaskan sebarang faedah perubatan atau kesihatan yang tertakluk kepada hak anda.

Data dan kerahsiaan

Segala maklumat anda yang diperolehi dalam penyelidikan ini akan disimpan dan dikendalikan secara sulit berdasarkan peraturan-peraturan dan/ atau undang-undang yang berkenaan. Data kajian ini akan digunakan untuk penghasilan laporan yang mungkin akan diterbitkan. Setelah semua data telah dikumpulkan dan penyelidikan diselesaikan, dokumen tersebut akan disimpan di dalam kabinet fail yang berkunci di pejabat penyelidik. Data akan disimpan di dalam komputer yang dikawal dengan

menggunakan kata laluan. Akses kepada data hanya boleh dilakukan oleh penyelidik. Selepas 5 tahun, dokumen tersebut akan dirincih dan dibuang. Data yang berkenaan akan dilaporkan secara kolektif tanpa merujuk kepada individu.

Pembayaran dan pampasan

Pembiayaan penyelidikan ini adalah ditanggung sendiri oleh penyelidik. Tiada pembiayaan luar atau geran yang diterima. Oleh itu, tiada bayaran yang akan diberi kepada anda untuk penyertaan anda dalam penyelidikan ini. Jika anda diperuntukkan ke dalam kumpulan 2 (kumpulan intevensi), anda juga tidak akan dikenakan caj bayaran bagi sesi terapi eksoskeletal robotik *Single Joint Hybrid Assistive Limb (HAL-SJ)* yang diterima.

Bolehkah penyelidikan ataupun penyertaan saya ditamatkan lebih awal daripada yang dirancang?

Penyelidik boleh menamatkan penyelidikan ini ataupun menamatkan penyertaan anda dalam penyelidikan ini pada bila-bila masa, jika perkara ini diperlukan demi keselamatan anda. Sekiranya penyelidikan ini dihentikan lebih awal atas sebab-sebab tertentu, anda akan dimaklumkan dan rawatan yang bakal anda terima selepas itu akan diuruskan. Anda juga akan diberi peluang untuk melihat keputusan penilaian anda pada rawatan susulan yang terakhir setelah tamat penyelidikan ini.

Siapakah yang perlu saya hubungi sekiranya saya mempunyai sebarang pertanyaan?

Sekiranya anda mempunyai sebarang pertanyaan mengenai penyelidikan ini ataupun anda rasa anda mempunyai kecederaan yang berkaitan dengan penyelidikan ini dan ingin memperolehi maklumat tentang rawatannya, sila menghubungi penyelidik beikut:

Dr. Chai Siaw Chui
Penyelia Utama
Program Terapi Carakerja
Fakulti Sains Kesihatan
Universiti Kebangsaan Malaysia
No. Telefon: 03-92897047
Emel: sc.chai@ukm.edu.my

Encik. Tan Eng Wah
Calon Sarjana
Program Terapi Carakerja
Fakulti Sains Kesihatan,
Universiti Kebangsaan Malaysia
No. Telefon: 012-6358531
Emel: tanew9911@gmail.com

BORANG PERSETUJUAN/ KEIZINAN PESERTA

Tajuk Penyelidikan: Keberkesanan Eksoskeletal Robotik Single Joint Hybrid Assistive Limb (HAL-SJ) dalam Meningkatkan Kefungsian di Kalangan Pekerja yang Mengalami Kepatahan Tulang Pergelangan Tangan: Percubaan Kawalan Rawak

Dengan menandatangani di bawah, saya mengesahkan bahawa:

- Saya telah diberi maklumat tentang penyelidikan di atas secara lisan dan bertulis. Saya telah membaca dan memahami segala maklumat yang diberikan dalam risalah ini.
- Saya telah diberikan masa yang secukupnya untuk mempertimbangkan penyertaan saya dalam penyelidikan ini dan telah diberi peluang untuk bertanyakan soalan dan semua persoalan saya telah dijawab dengan sempurna dan memuaskan.
- Saya juga faham bahawa penyertaan saya adalah secara sukarela dan pada bila-bila masa saya bebas menarik diri daripada penyelidikan ini tanpa harus memberi sebarang alasan dan ianya sama sekali tidak akan menjejaskan rawatan perubatan saya pada masa akan datang. Saya tidak mengambil bahagian dalam mana-mana penyelidikan lain pada masa ini. Saya juga memahami tentang risiko dan manfaat penyelidikan ini dan saya secara sukarela memberi persetujuan untuk menyertai penyelidikan ini di bawah syarat-syarat yang telah dinyatakan di atas. Saya faham bahawa saya harus mematuhi nasihat dan arahan daripada penyelidik berkaitan dengan penyertaan saya dalam penyelidikan ini.
- Saya faham bahawa segala maklumat dan data peribadi akan dianggap sebagai SULIT.
- Saya akan menerima satu salinan ‘Risalah Maklumat Peserta dan Borang Persetujuan atau Keizinan Peserta’ yang telah lengkap dengan tarikh dan tandatangan untuk dibawa pulang ke rumah.

Subjek:

Tandatangan: Nombor K/P:

Nama: Tarikh :

Penyelidik yang mengendalikan proses menandatangani borang keizinan:

Tandatangan: Nombor K/P:

Nama: Tarikh :

Saksi tidak-berpihak/adil:

Tandatangan: Nombor K/P:

Nama: Tarikh :

Appendix L: SOCSO TRRC Scientific Committee Approval Letter

PUSAT REHABILITASI PERKESO SDN BHD. 1122345[illegible]
PERTUBUHAN KESELAMATAN SOSIAL
(Socia) Sec11o1y Organisation)
Lot F'f7263 111.S (D) 189230
Hang Tuah Jaya, Bandar Hijau,
75450 Melaka
No.Tel.: 1606 235 4000
No.Faks: [illegible]606 253 5150
Laman web : www.rehabmalaysia.co111
E-mel : [illegible]

Rujukan Tuan :
Rujukan Kami : PRPTAR.600-5148)
Tarikh : 11thNovember 2020

Mr. Tan Eng Wah
Faculty of Health Sciences,
Universiti Kebangsaan Malaysia
(UKM), 43600 Bangi, Selangor.

Dear Sir,

STATUS OF APPROVAL OF RESEARCH PROPOSAL: THE EFFECTIVENESS OF SINGLE JOINT HYBRID ASSISTIVE LIMB (HAL-SJ) ROBOTIC EXOSKELETON IN IMPROVING FUNCTIONAL OUTCOMES AMONG WORKERS WITH WRIST FRACTURES: A RANDOMIZED CONTROLLED TRIAL IN SOCSO TUN RAZAK REHABILITATION CENTRE.

I would like to refer to the above mentioned matter.

Research Title : The Effectiveness Of Single Joint Hybrid Assistive Limb (HAL-SJ) Robotic Exoskeleton In Improving Functional Outcomes Among Workers With Wrist Fractures: A Randomized Controlled Trial

Researcher : Mr. Tan Eng Wah

2. I am pleased to inform you that the Research Committee, Research and Development Department, SOCSO Rehabilitation Centre had approved in principle, the study research of the above project.

3. With respect to the research proposal, the researcher is allowed to conduct the data collection and intervention procedures on the targeted subjects, who are the SOCSO insured persons, without any financial obligation.

4. The timeline of the study is as per the Gantt-Chart attached with the proposal.

Thank you

"Look at the Ability, Not the Disability"

Yours sincerely,

(DR HAFEZ BIN HUSSAIN)
Chairman
Research & Development Department
Pusat Rehabilitasi PERKESO Sdn. Bhd.

"Look at the Ability, Not the Disability"
